# Nanocarriers for effective delivery: modulation of innate immunity for the management of infections and the associated complications

**DOI:** 10.1186/s12951-022-01582-8

**Published:** 2022-08-19

**Authors:** Chung-Nga Ko, Shaohong Zang, Yingtang Zhou, Zhangfeng Zhong, Chao Yang

**Affiliations:** 1 National Engineering Research Center for Marine Aquaculture, Institute of Innovation & Application, Zhejiang Ocean University, Zhoushan, 316022 Zhejiang China; 2 C-MER Dennis Lam and Partners Eye Center, Hong Kong International Eye Care Group, Hong Kong, China; 3 C-MER (Shenzhen) Dennis Lam Eye Hospital, Shenzhen, Guangdong China; 4 C-MER International Eye Research Center of the Chinese University of Hong Kong (Shenzhen), Shenzhen, China; 5 Macau Centre for Research and Development in Chinese Medicine, Institute of Chinese Medical Sciences, University of Macau, Taipa, 999078 Macao SAR China

**Keywords:** Innate immunity, Nanotechnology, Immunotherapy, Immunomodulator, Infectious diseases, Sepsis

## Abstract

Innate immunity is the first line of defense against invading pathogens. Innate immune cells can recognize invading pathogens through recognizing pathogen-associated molecular patterns (PAMPs) via pattern recognition receptors (PRRs). The recognition of PAMPs by PRRs triggers immune defense mechanisms and the secretion of pro-inflammatory cytokines such as TNF-α, IL-1β, and IL-6. However, sustained and overwhelming activation of immune system may disrupt immune homeostasis and contribute to inflammatory disorders. Immunomodulators targeting PRRs may be beneficial to treat infectious diseases and their associated complications. However, therapeutic performances of immunomodulators can be negatively affected by (1) high immune-mediated toxicity, (2) poor solubility and (3) bioactivity loss after long circulation. Recently, nanocarriers have emerged as a very promising tool to overcome these obstacles owning to their unique properties such as sustained circulation, desired bio-distribution, and preferred pharmacokinetic and pharmacodynamic profiles. In this review, we aim to provide an up-to-date overview on the strategies and applications of nanocarrier-assisted innate immune modulation for the management of infections and their associated complications. We first summarize examples of important innate immune modulators. The types of nanomaterials available for drug delivery, as well as their applications for the delivery of immunomodulatory drugs and vaccine adjuvants are also discussed.

## Introduction

Infection occurs when germs e.g. bacteria or viruses enter human body, increase in number, and cause harm. The classical symptoms of bacterial and viral infections include fever, headache, skin flushing, and fatigue etc. [1]. When invasion occurs, the body’s immune system has two fundamental lines of defense against invading pathogens, known as (1) innate immunity and (2) adaptive immunity [2]. Innate immunity, also known as natural immunity, is the first line of defense against invading pathogens [3]. At the cellular level, innate immune response is mediated mainly by innate immune cells including macrophages, monocytes, and neutrophils. At the molecular level, innate immune cells sense invading pathogens through recognition of pathogen-associated molecular patterns (PAMPs), such as bacterial membrane components (e.g. peptidoglycans and lipopolysaccharides (LPS)), bacterial/viral genome and envelope proteins, by pattern recognition receptors (PRRs) [4]. The recognition of PAMPs by PRRs triggers immune defense mechanism and the secretion of pro-inflammatory cytokines such as TNF-α, IL-1β, and IL-6, which alerts the nearby cells to the presence of invaders and recruit more immune cells to the infection site [5]. The activation of PRRs also stimulates microbicidal mechanism of phagocytic cells, leading to the generation of toxic oxidants (e.g. reactive oxygen species (ROS) and reactive nitrogen species (RNS) etc.) and antimicrobial peptides [6]. On the other hand, adaptive immunity is mediated by B cells and T cells in response to the recognition of foreign antigens and the innate immune response [7, 8]. Inflammatory response is a complex but coordinated process. Once the infection is under control, anti-inflammatory cytokines are produced to alleviate the immune response and reverse the immune system to normal homeostasis [9].

However, excessive activation of immune system may disrupt immune homeostasis and consequently contributes to serious medical conditions. For example, sepsis is a life-threatening systemic inflammatory syndrome due to a dysregulated host response to infection [10, 11]. At the early stage of sepsis, inflammatory response becomes incredibly powerful and uncontrollable, leading to the overproduction of pro-inflammatory cytokines and the induction of “cytokine storm” [12]. If left untreated, apoptosis of immune cells (e.g. macrophages, lymphocytes, dendritic cells, B cells, and T cells etc.) could induce immunosuppression and cause a secondary bacterial infection, which may lead to irreversible damage of tissues, multiple organ failure and even death [13]. Although immune cell apoptosis is a crucial event in the pathogenesis of sepsis-induced immunosuppression, the detailed molecular mechanisms are still not fully understood. It has been reported that the (1) extrinsic death receptor pathway, (2) mitochondrial pathway, and (3) endoplasmic reticulum (ER) stress-induced pathway are involved in immune cell apoptosis [14, 15]. For example, mitochondrial pathway can be mediated by Bcl-2 family proteins including the anti-apoptotic proteins (e.g. Bcl-2, Bcl-xL and Bcl-w) and the proapoptotic proteins (e.g. Bad, Bmf, Bak, Bid and Bim) [16]. In septic patients, the proapoptotic proteins such as Bak, Bim and Bid were massively upregulated, whereas the anti-apoptotic protein Bcl-2 was found to be downregulated. To date, the global burden of sepsis remains uncertain due to the fact that most sepsis-related deaths may not be classified as being caused by sepsis [17]. A systemic review recently estimated that there are approximately 6 million sepsis-related deaths worldwide every year. However, this estimate did not include the statistics from the low- and middle-income countries, where over 85% of global population lives, making it difficult to estimate the true burden of sepsis [18].

Nowadays, antibiotics have been commonly used for the treatment of bacterial infections [19, 20]. However, the sustained and indiscriminate use of antibiotics has contributed to the emergence of antimicrobial-resistant (AMR) strains. In 2016, the Centers for Disease Control and Prevention (CDC) estimated that 20–50% of antibiotics prescribed in acute care hospitals in the United State are inappropriate and unnecessary. It is also estimated that over 70% of bacteria that cause bacterial infection in the United State are resistant to at least one commonly used antibiotic [21]. In spite of the fact that viruses cannot be killed by antibiotics, collected data shows that the problem of AMR is somewhat exacerbated by the COVID-19 pandemic as most of the patients hospitalized for COVID-19, even without critical illnesses, received antibiotics [22]. At present, AMR-associated infections claim over 700,000 human lives worldwide annually and the number are projected to rise markedly to over 10 million after 20 years [23]. Although substantial efforts have been made to the discovery of new antibiotics for treating infectious diseases, the discovery rate of new antibiotics could never keep up with the ever-changing evolution of resistant strains [24]. More importantly, antibiotics usually share similar mechanism of action, making the emergence of AMR strains inevitable [25, 26]. The World Health Organization (WHO) declared in 2020 that AMR will remain one of the ten biggest threats to public health in the world [27]. On the other hand, treatments of viral infections have proven to be challenging due to their tiny size and the ability to reprogram human cells for self-reproduction [28]. To date, treatments of viral infections mostly focus on symptom relief, and patients usually need to wait for their own immune system to fight off the virus [29, 30]. To alleviate the global burdens of infections, one alternative over the conventional strategies is to target the host–pathogen interface [31].

Immunotherapy is a type of treatment harnessing the power of immune system to fight against diseases, such as infectious diseases, inflammatory diseases, and cancers [32]. To date, hundreds of new and promising immunotherapeutic options have been available in clinical trials for different stages of cancers. As of December 2021, the US Food and Drug Administration (FDA) has approved over thirty immunotherapies for the treatment of cancers such as lung cancer, prostate cancer, gastric cancer, bladder cancer, melanoma, and lymphoma [33, 34]. Importantly, infectious diseases, inflammatory diseases and cancers have a shared hallmark of immune dysregulation. Therefore, immunotherapy may also be beneficial to treat infectious diseases and their associated complications. Recently, a range of immunomodulators for PRRs (e.g. toll-like receptors and NOD-like receptor) signaling and innate defense regulator (IDR) peptides, have been reported [35–38]. However, their therapeutic performances can be negatively affected by 1) high immune-mediated toxicity, 2) poor solubility and 3) bioactivity loss after long circulation. Recently, nanocarriers have emerged as a very promising tool to overcome these obstacles owning to their unique properties such as sustained circulation, desired bio-distribution, and preferred pharmacokinetic and pharmacodynamic profiles. For example, nanocarriers can readily improve the safety and efficacy of immunomodulators by lowering their toxicity and/or enhancing their specificity. Compared to free immunomodulators, nanocarrier-assisted approaches generally have improved therapeutic performances in vivo [39].

In this review, we aim to provide an up-to-date overview on the strategies and applications of nanocarrier-assisted innate immune modulation for the management of infections and the associated complications. We first summarize examples of important innate immune modulators, including PRRs signaling and some innate defense regulators, as well as the importance of some innate immune cells. The types of nanomaterials available for drug delivery, as well as their applications for delivery of immunomodulatory drugs and vaccine adjuvants are also discussed. However, immunomodulatory approaches for adaptive immunity are out of the scope and are not discussed in this review.

## Modulation of innate immune response

Innate immune response is the first line of defense against invading pathogens. However, if immune response becomes uncontrollable, the overproduction of pro-inflammatory cytokine could contribute to inflammatory disorders. Understanding the role of innate immune system and the pathogenesis of infections are crucial for timely and appropriate clinical management of the diseases.

Innate immune modulators are often used as therapeutic agents or vaccine adjuvants [40]. When used as therapeutics, immunomodulators may be used alone or used in conjunction with antibiotics. However, how to avoid the protective immune response from turning into harmful inflammatory diseases remains one of the main challenges. The potential value of IDR peptides in the treatment of infectious diseases has raised considerable attention in recent years. Animal studies have shown that IDR peptides may possibly stimulate protective immune system without causing excessive inflammatory responses. In this section, we summarize a range of PRRs-targeting immunomodulators, examples of IDR peptides, and the importance of certain innate immune cells, for the management of infectious diseases and the associated complications.

### Pattern recognition receptors (PRRs)

PRR is a class of receptors that recognize PAMPs, apoptotic host cells, and damaged senescent cells. In general, innate immune response is modulated by the collaboration between multiple PRRs, such as Toll-like receptors (TLRs) and NOD-like receptors (NLRs), as well as their downstream mediators. Increasing evidence has suggested the importance of PRRs in the management of both infectious and inflammatory diseases. For instance, it has been found that Asp299Gly and Thr399lle SNPs of TLR4 are associated with an increased risk of gram-negative bacterial infections and sepsis [41]. A study conducted by Theo et al. shows that polymorphism of TLR1 increases susceptibility to *Candidemia* [42], which is a severe infection caused by a pathogenic yeast called *Candida albicans*. Further studies have demonstrated that polymorphism of TLR4 is associated with impaired control of bacterial infections in human [43].

PRRs are mainly expressed in antigen presenting cells such as macrophages and dendritic cells. Depending on the type of PRRs, they are located either on cell membrane, or are distributed in cytoplasm or intracellular compartment membranes [44]. Modulation of PRRs can be driven by the use of molecules that mimic the structure of natural ligands. Agonists or antagonists could either activate or suppress the signaling pathways of PRRs and mediate different effects: (1) release or suppression of pro-inflammatory cytokines and chemokines; (2) formation or inhibition of an inflammatory microenvironment; (3) induction or inhibition of chronic inflammation etc. Other innate immune PRRs also include retinoic acid-inducible gene (RIG)-like receptors, but their role in the pathogenesis of infections have not yet been formally studied [45]. To date, pharmaceutical companies have developed a range of chemical-, aptamer-, peptide-, protein-based immunomodulators targeting various PRRs, which can be categorized into membrane-bound, endosomal, and cytoplasmic subgroups.

#### Modulation of plasma membrane bound PRRs signaling

Recent studies have shown that TLRs are involved in the mediation of systemic responses to pathogens and sepsis. To date, at least 6 plasma membrane bound TLRs, including TLR1, TLR2, TLR4, TLR5, TLR6, and TLR11, have been identified. These transmembrane receptors consist of an extracellular domain that interacts with PAMPs and an intracellular domain for signal transmission. A majority of these TLRs recognize molecular structures of microbial proteins (e.g. flagellin from bacterial flagella), LPS from Gram-negative bacteria, lipoteichoic acid (LTA) and peptidoglycan (PGN) from Gram-positive bacteria, and lipoglycans, lipopeptides and lipomannans from mycobacteria etc.

**Fig. 1:**
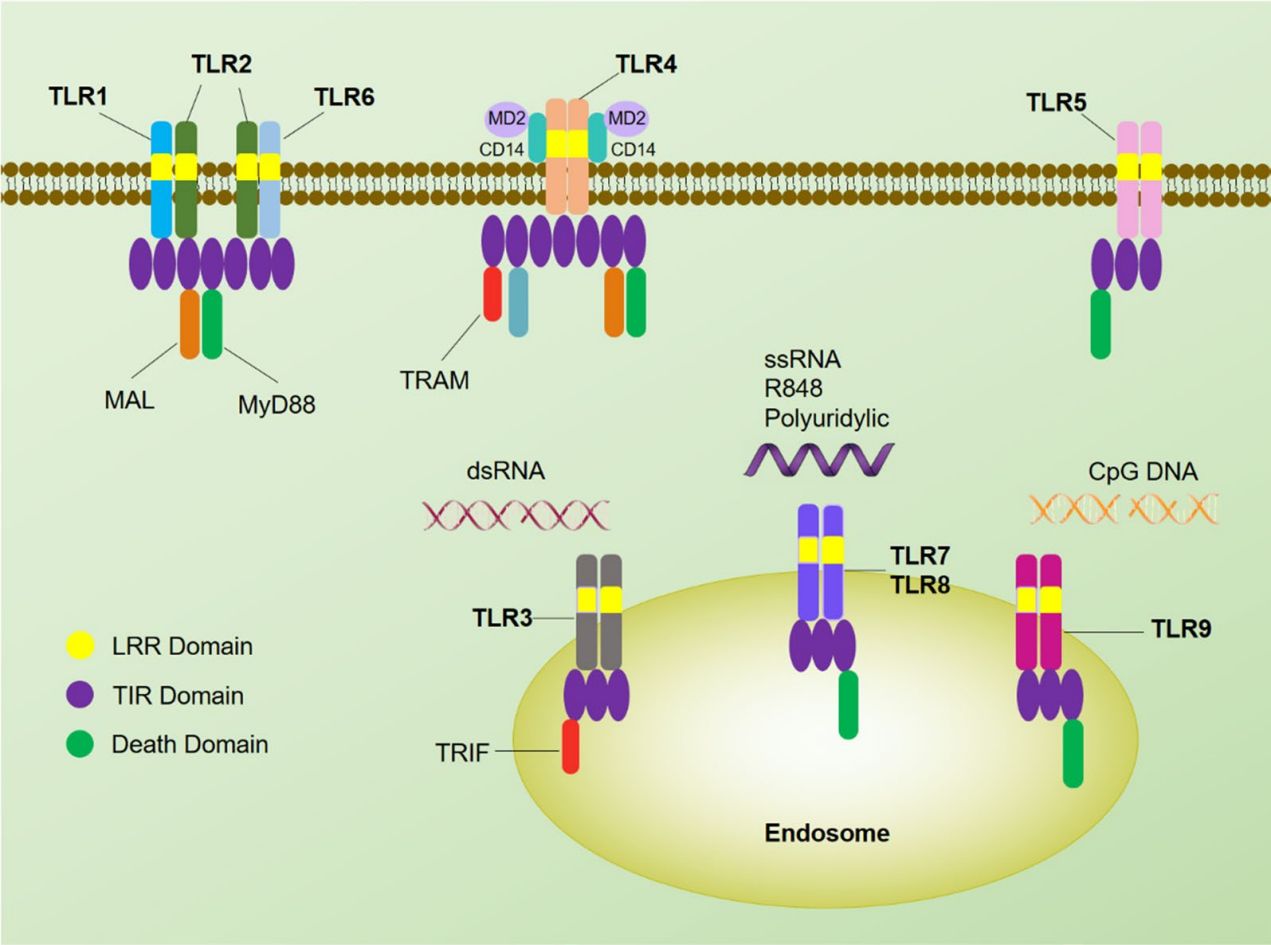
TLRs of innate immune system and their signaling pathways

Upon recognizing PAMPs on cell surface, nuclear factor-κB (NF-κB) and mitogen-activated protein kinase (MAPK) are activated through TIR domain adaptor molecule (TRAM), TIR domain-containing adaptor protein (TIRAP), TIR domain-containing adaptor protein inducing IFN-β (TRIF), TIR domain containing adaptor protein (myeloid differentiation 88 (MYD88)) and other downstream mediators (Fig. 1). The signaling process ultimately drives the production of pro-inflammatory cytokines and chemokines. Except for TLR4 that signals through both MYD88-dependent and TRIF-dependent pathways, all plasma membrane bound TLRs signal through MYD88-dependent pathways [46]. To date, various TLRs have been recognized as the molecular targets for the treatment of infectious diseases and sepsis. For example, Luivac, an oral immunomodulator comprising seven bacterial species, is often used for minimizing the risk of recurrent respiratory tract infections in children, potentially through the activation of various TLRs [47].

##### TLR1, 2, 6

TLR2 recognizes diverse bacterial structures, such as lipopeptides, peptidoglycan, and LTA derived from Gram-positive bacteria, as well as molecular patterns of viruses such as viral envelope protein. TLR2 can sense a variety of ligands because of its ability to heterodimerize with either TLR1 or TLR6 on cellular surface. The TLR2/1 complex is able to recognize triacylated lipopeptides, while the TLR2/6 complex is able to sense diacylated lipopeptides. Although studies have shown that TLR2 is able to form a heterodimeric complex with TLR10, no defined ligands have been identified for TLR2/10 complex so far. The use of TLR2 as therapeutic targets against infections and sepsis have been summarized in a recent review [48]. It has been reported that TLR2 agonist offers protection against *methicillin-resistant Staphylococcus aureus* infection through enhancing the bactericidal activity of neutrophils in mice model. Recent evidence also suggests that TLR2 may be one of the major contributors to the pathogenesis of sepsis. For example, Bergt et al. reported that TLR2-deficient mice had markedly improved survival during sepsis [49]. Lima et al. also reported that inhibition of TLR2 through administration of anti-TLR2 antibody relieved systemic inflammation in mice models with polymicrobial sepsis [50]. Apart from therapeutic purpose, TLR2 are also being investigated as adjuvants to boost efficacy of vaccines. Table 1 summarizes the recent clinical trials, both completed and ongoing trials, of a series of TLR-based therapies or vaccine adjuvants. For example, XS15, which is a synthetic TLR1/2 ligand, has been developed as an adjuvant for peptide vaccination. The use of XS15 as a TLR agonist adjuvant is currently in Phase I clinical trial for improving the efficacy of SARS-CoV-2 vaccine in adults.

**Table 1:** A summary of immunomodulators being investigated in the clinical stage and their applications for the management of infections and sepsis

Drug	PRR target	Type	Role	Application	Phase of development	Participants	Status	References
imiquimod	TLR7 agonist	Small molecule	Therapeutics	Genital warts (caused by HPV)	Clinical	/	FDA approved	/
Luivac (LW 50,020)	Agonist for various TLRs	Bacterial lysates	Therapeutics	Respiratory tract infections	Clinical	33	Completed	[47]
MGN1703	TLR9 agonist	Synthetic DNA	Adjuvant	HIV	Phase 1b/2a	12	Completed	NCT02443935
CPG 7909	TLR9 agonist	Synthetic DNA	Therapeutics	HIV	Phase 1	97	Completed	NCT00562939
XS15	TLR1/2 ligand	Synthetic Pam3Cys-derivative	Adjuvant	SARS-CoV-2	Phase 1	36	Active but not recruiting	NCT04546841
SD-101	TLR9 agonist	Synthetic oligonucleotide with CpG motifs	Adjuvant	HCV	Phase 1	34	Completed	NCT00823862
GS-9620	TLR7 agonist	Small molecule	Therapeutics	HBV	Phase 1	51	Completed	NCT01590654
imiquimod	TLR7 agonist	Small molecule	Adjuvant	Influenza virus	Phase 3	160	Completed	NCT02103023
resiquimod	TLR7/8 agonist	Small molecule	Adjuvant	Influenza virus	Phase 1	59	Completed	NCT01737580
eritoran	TLR4 antagonist	Synthetic lipid A antagonist	Therapeutics	Sepsis	Phase 3	197	Completed	NCT00334828
TAK-242	TLR4 antagonist	Small molecule	Therapeutics	Sepsis	Phase 3	277	Completed	NCT00143611
NI-0101	TLR4 antagonist	Monoclonal antibody	Therapeutics	Sepsis	Phase 1	80	Completed	NCT01808469
RO7020531	TLR7 agonist	Small molecule	Adjuvant	HBV	Phase 1	46	Completed	NCT02956850
VAX125	Consist of a TLR5 ligand	A recombinant hemagglutinin influenza-flagellin fusion	Adjuvant	Influenza	Phase 2	128	Completed	NCT00730457
imiquimod	TLR7 agonist	Small molecule	Adjuvant	HBV	Phase 2	100	Active but not recruiting	NCT04083157
polyI:C	TLR3 agonist	Synthetic analog of dsRNA	Adjuvant	SARS-CoV-2	Phase 1	48	Not yet recruiting	NCT05155982
IMO-2125	TLR9 agonist	Synthetic DNA	Therapeutics	HCV	Phase 1	63	Completed	NCT00990938
IMO-2125	TLR9 agonist	Synthetic DNA	Therapeutics	HCV	Phase 1	58	Completed	NCT00728936

##### TLR4

TLR4, a TLR predominantly expressed in myeloid, recognizes molecules from gram-negative bacteria (e.g. LPS). Studies have shown that TLR4, when transfected alone, is not enough for recognizing LPS [51]. However, when physically associated with its co-receptor myeloid differentiation 2 (MD-2), the TLR4-MD-2 complex could specifically recognize LPS molecule [52]. Other accessory molecules e.g. CD14 and LPS-binding protein (LBS) also facilitate the binding of LPS with TLR4-MD2. Monophosphoryl lipid A (MPLA), a TLR4 agonist, is a non-toxic derivative of the lipid A portion of bacterial LPS endotoxin [53]. While maintaining similar immunostimulatory effect to LPS, MPLA displays at least a 100-fold lower toxicity. In 1986, Travenol et al. reported that MPLA could significantly improve the survival rates of the treated animals infected with either *E. coli* or *S. epidermidis* [54]. In a later study, Takashi et al. found that MPLA is able to improve the clearance of *Moraxella catarrhalis* and *Haemophilus influenzae* in mice through TLR4 activation [55]. Recent studies also illustrated that administration of LPS (a TLR4 agonist) at the time of infection offers partial protection against *Bordetella pertussis* in mice [56].

While TLR-dependent signaling enables protective innate immune response, sustained and overwhelming activation of TLRs may disrupt immune homeostasis and contribute to the development of sepsis and other inflammatory diseases. Over the past years, numerous TLR antagonists have been developed for the treatment of sepsis. For example, eritoran tetrasodium (E5564), a TLR4 antagonist, is able to limit excessive LPS-induced inflammation in animal models by competitively binding to TLR4-MD2 [57]. However, despite the encouraging results in animal studies, a phase III clinical study suggests that E5564 could not confer survival benefits to patients with severe sepsis [58]. Other TLR4 antagonists also include TAK-242, Opioids, Ketamine and Lansoprazole etc. However, recent clinical trials using these immunomodulators on patients with sepsis had disappointing results. For example, a clinical trial of 274 patients shows that TAK-242 did not suppress the level of cytokine expression in patients with sepsis [59]. Zhang et al. also evaluated the survival differences between patients treated with opioid and placebo among 6000 patients with sepsis. The result surprisingly shows that the opioid-treated patients had a significantly higher mortality rate compared to the non-treated patients [60]. Although the role of TLR4 in the development of sepsis remains debatable, investigations using TLR4 antagonists for the treatment of sepsis is still ongoing. Modulators of TLR4 are also being investigated as adjuvants to boost the efficacy of vaccines (Table 1). For example, AS04 adjuvant, which is a combination of MPL (a TLR4 agonist) and aluminum salt, induces production of co-stimulatory molecules e.g. cytokines and chemokines [61]. AS04-adjuvated HPV 16/18 vaccine, also known as Cervarix, received FDA approval in 2009 for the prevention of cervical cancer caused by HPV infection.

##### TLR5

TLR5 is preferentially expressed in respiratory epithelial cells and is known to sense flagellin specifically. For example, TLR5 found on airway epithelium can recognize *Pseudomonas aeruginosa,* which is a Gram-negative bacterium that can cause severe infection [62]. TLR5 is also expressed in intestinal epithelial cells for recognizing bacteria that flow across the gut [63]. Studies have shown that administration of flagellin (a TLR5 agonist), either concurrently or after bacterial infection, offers protection against infections with *Pseudomonas aeruginosa* and *Streptococcus pneumoniae* [64]. A Phase III clinical trial shows that VAX125, a recombinant influenza vaccine consisting of flagellin (a TLR5 agonist), elicits vigorous responses against native virions at a relatively low dose of influenza hemagglutinin antigen, suggesting the substantial adjuvant effect of flagellin [65, 66].

#### Modulation of endosomal PRRs signaling

In contrast to plasma membrane bound PRRs that mainly recognize PAMPs on the surface of bacteria (e.g. lipopeptide, lipoprotein, PGN and LPS from bacteria), endosomal PPRs (i.e. TLR3, TLR7, TLR8, TLR9, TLR12 and TLR13) primarily sense bacterial nuclear components and virus-derived nucleic acids, which gains access to the endosomal compartment of cells by phagocytosis of apoptotic cell debris or pathogen-infected cells [67, 68]. While all the endosomal TLRs signal through MYD88 signal pathway, TLR3 exclusively signals through TRIF-dependent pathway [69]. In this section, the preliminary results and clinical uses of some important endosomal TLRs in various infectious diseases are discussed.

##### TLR3

TLR3 preferentially detect dsRNA from a range of viruses. Signal of TLR3 is transduced through the TRIF-dependent pathway, which ultimately leads to the induction of IFN responses, as well as the production of inflammatory cytokines [70, 71]. TLR3 can specifically recognize RNA viruses including West Nile, influenza A, and rhinovirus etc. [72, 73]. It has also been found that TLR3 can recognize certain types of DNA viruses e.g. herpes simplex virus 1 (HSV-1) [74]. Recent evidence has shown that TLR3-deficient mice are more susceptible to viral infections, both ssRNA and DNA viruses [75]. Polyinosinic-polycytidylic acid (poly I:C) is a synthetic TLR3 agonist [76]. Intranasal administration of Poly I:C within 48 h post-infection was found to offer protection to mice with lethal respiratory viral infection [77]. Other studies have shown that Poly I:C was able to inhibit replication of SARS-CoV-2 in mice, with an inhibitory effect higher than that of chloroquine and nelfinavir [78]. The use of Poly I:C as potent vaccine adjuvants has also been investigated for years. For example, TriAdj, an adjuvant consisting of poly(I:C) and IDR-002 (an immunostimulatory HDP), has been developed for boosting the efficacy of different vaccines [79]. Most recently, TriAdj has entered Phase I clinical trial for improving the immunogenicity response to the S1 protein of SARS-CoV-2 [80].

##### TLR7

TLR7 preferentially binds to viral ssRNA and has been shown to play an important role in protecting the body against viral infections [81]. Imidazoquinoline and imiquimod are synthetic oligonucleotides that mimic viral components, activate TLR7, and induce the production of pro-inflammatory cytokines [82]. Studies have shown that TLR7-deficient mice are not able to respond to ssRNA of viruses including VSV, HIV-1 and IAV etc. [83]. Recently, topical application of imiquimod has been proven to be safe and effective for treating external anogenital warts, which is a type of infection commonly caused by HPV [84]. Furthermore, topical application of imiquimod has been shown to boost the efficacy of influenza vaccine in animal model and has successfully completed a Phase III clinical study in young healthy individuals and elderly [85].

##### TLR9

TLR9 specifically recognizes non-methylated CpG DNA from bacteria and viruses. Upon engaging with CpG DNA, TLR9 is activated and can transmit signal through MDY88-dependent pathway, which consequently initiate innate immune response [71]. TLR9 is a known sensor for a range of DNA viruses including HSV-1, HSV-2, cytomegalovirus (MCMV), and adenovirus. Studies have shown that administration of TLR9 agonists could reduce viral load and improve the survival of mice infected with HSV-1 encephalitis (HSE) [86]. CpG ODN was found to inhibit the replication of HBV in infected murine [87]. CPG10101, a TLR9 agonist, was shown to elicit a dose-dependent enhancement of immune response and lower the level of HCV RNA in a Phase I clinical trial [88]. However, interactions between pathogens and hosts are likely to be complicated, and the outcomes may be difficult to predict. For example, Wang et al. reported the use of a CpG ODN and imiquimod as immunomodulators for preventing transmission of simian immunodeficiency virus (SIV) [89]. While both ODN and imiquimod could induce innate immune response in the cervicovaginal mucosae of rhesus monkeys, all treated monkeys become infected after SIV inoculation, and had a higher level of plasma viral RNA compared to that of the control monkeys. On the other hand, immune activation by CpG ODNs was found to be beneficial as vaccine adjuvants [90]. Etsuro et al. evaluated various formulations of PRR agonists, and found that vaccine adjuvants that consists of CpG ODN and aluminum hydroxide had a substantially higher immune response toward SARS-CoV-2 receptor binding domain in aged mice [91]. In clinical practice, CpG-7909, which is a synthetic CpG ODN, has been proven to be a safe and effective adjuvant for improving the efficacy of the anthrax vaccine BioThrax [92].

#### Modulation of cytoplasmic innate immune signaling

When pathogens access the interior of a host cell, PAMPs of pathogens, particularly peptides, can be detected by cytoplasmic PRRs such as NOD proteins [93]. In this section, NOD-like receptors (NLRs) and stimulator of interferon genes (STING), which are among the most prominent pathways that respond to microbials in cytoplasm, are discussed.

##### NOD-like receptors (NLRs)

NLRs are a family of cytoplasmic receptor recognizing PAMPs that gain access to the interior of cells via phagocytosis, as well as damage-associated molecular patterns (DAMPs) released from dying or damaged cells due to pathogenic infections [94, 95]. NLRs can be categorized into two sub-families i.e. NODs and NLRPs subfamilies. Among all the NLRs, nucleotide-binding oligomerization domain-containing 1 (NOD1), NOD2 and NOD-, LRR- and pyrin domain-containing 3 (NLRP3) are the most well-characterized members of the family, and are responsible for recognizing microbial signals [96, 97]. For example, NOD1 specifically senses muropeptides from Gram-negative bacteria that contains diaminopimelate (DAP), while NOD2 detects muramyl dipeptide (MDP) that can be found in all kinds of bacteria [98]. NOD1 and NOD2 can be activated through ligand recognition, which results in the activation of MAPK and NF-κB signaling and the secretion of pro-inflammatory cytokines and chemokines [99]. On the other hand, NLRP3 recognize signals from both PAMPs and DAMPs, which consequently induce the NLRP3 inflammasome-dependent immune responses and facilitate the removal of invading microbials [100]. To date, numerous immunomodulators targeting NLRs have been developed [101]. Evidence has suggested that mice pretreated with NOD-1 ligands orally or parentally prior to bacterial infections had higher survival rate. Compared to treatments using antibiotics alone, treatments using both immunomodulatory muropeptides and antibiotics were found to offer greater resistance to bacterial infections [45]. Furthermore, murabutide, which is a synthetic derivative of MDP (a NOD2 agonist), has successfully completed a Phase II clinical trial demonstrating its clinical tolerance and potential for improving the antiviral immunity in HIV-infected patients [102].

Other than agonistic studies, antagonistic studies which focus on the blockage of NLR function are also advantageous. It has been found that the activities of NOD1 and NOD2 are upregulated during viral infections [103]. Secondary bacterial infections that manifest during or after a viral infection may trigger uncontrolled inflammatory responses and lethality. A potent NOD antagonist for inhibition of NOD activities may blunt the associated pro-inflammatory responses and decrease the rate of mortality. Recently, some dual NOD1/NOD2 antagonists that demonstrate promising inhibitory activities against NOD1/NOD2-stimulated NF-κB and MAPK signaling have been developed [104, 105]. However, these compounds are still at the preclinical investigation stage, and none have shown promising activities against infection-associated complications.

##### Stimulator of interferon genes (STING)

STING is a cytosolic sensor responsible for regulating innate immune defense. In mammalian cells, the detection of foreign DNA (e.g. microbial DNA), is mainly contributed by the cyclic GMP–AMP synthase (cGAS)–STING pathway. The binding of cGAS to cytosolic microbial dsDNA species, such as HSV-1 and HBV, allosterically activates its catalytic activity and leads to the production of cyclic dinucleotides (CDNs), which is a secondary messenger molecule and potent agonist of STING. The sensing of CDNs induces a conformational change in STING and triggers the production of NF-κB- and IRF3-dependent cytokines. Following these events, STING is rapidly degraded to avoid excessive production of cytokines [106, 107]. On the other hand, some intracellular bacteria (e.g. *Listeria monocytogenes*) can generate CDNs that directly promote the activation of STING-dependent signaling pathway [108]. However, some DNA viruses and bacteria have been found to develop strategies to disrupt the pathway. For example, E7, which is a HPV-encoded product, is able to bind with STING and abrogate the cGAS-STING signaling pathway [109]. HSV-1 is also known to encode at least nine products, such as ICP27, VP22, UL24 and UL46, to suppress the signaling of cGAS-STING-mediated pathway [110]. It has also been reported that STING or cGAS knockout mice were more susceptible to infections by HSV1 and other DNA viruses, suggesting the importance of STING-dependent pathway in host defense mechanism against microbial infections [111].

To date, numerous STING agonist have been developed. For example, 5,6-dimethylxanthenone-4-acetic acid (DMXAA), a STING agonist, has been reported to induce the production of IFNs in mice and reduce the viral load of HSV-1 in the peripheral and central nervous systems [112]. DMXAA was also found to induce antiviral innate immunity and suppress the replication of HBV in mice [113]. More recently, dimeric amidobenzimidazole (diABZI), which is a synthetic small molecule of STING agonist, was also found to activate and limit the replication of SARS-CoV-2 and HCoV-229E in cells and animals effectively [114, 115]. These findings highlight the therapeutic potential of STING-dependent pathway in the treatment of infectious diseases.

### Innate defense regulator (IDR) peptides

IDR peptides, traditionally known as antimicrobial peptides (AMP), are synthetic compounds that possess both anti-infective and immunomodulatory effects. The amino acid compositions of IDR peptides are designed based on the sequences of natural host defense peptides (HDPs), which are produced by innate immune cells against bacterial challenges. The strategies for designing IDR peptides have been summarized and reviewed by Donna et al. [116]. While some HDPs (e.g. α-defensins) possess weak direct microbicidal effects in vivo, most HDPs eliminate infections through mediating immunomodulatory activities on the host. Intriguingly, HDPs are known to induce the production of pro-inflammatory cytokines while promoting a local non-inflammatory resolution of infections. For example, Roel et al. reported that CATH-2, a type of Cathelicidins HDP, could efficiently neutralized the activation of LPS-induced M1 macrophage [117].

To date, many IDR peptides have been developed and employed in antimicrobial therapies. IDR-1, which is one of the first synthetic IDR peptides used for treating bacterial infection, has been shown to mediate protection against bacterial infections such as vancomycin-resistant *Enterococcus* and methicillin-resistant *Staphylococcus aureus* in mice without causing obvious cytotoxicity [118]. Numerous IDR peptides that possess both immunostimulatory and anti-infective activities have entered clinical stage (Table 2). For example, a pre-clinical investigation shows that EA-230, a synthetic IDR peptide, is effective in treating infection-induced inflammatory diseases [119]. EA-230 was also found to confer survival benefits in animals with abdominal sepsis [119]. Furthermore, EA-230 demonstrated excellent safety and tolerability in healthy volunteers participating in Phase I clinical trials [120]. IMX-942, a synthetic IDR peptide designed based on the sequence of IDR-1, first entered Phase I clinical trial in 2009 [116]. IMX-942 has no direct anti-infective activities. However, IMX-942 is able to modulate innate immune response by binding with adaptor protein and changing the downstream signaling network of TLRs and TNF [121]. Other IDR peptides that possess promising pre-clinical data also include IDR-1002, IDR-HH2 and IDR1018, which were found to reduce inflammation in mice infected with *Pseudomonas aeruginosa* [122] and *Mycobacterium tuberculosiss* [123].

**Table 2:** A summary of IDR peptides that have entered clinical stage for the treatment of bacterial infections and sepsis

Drug	Description	Mechanism	Route of Administration	Potential clinical application	Phase of development	Participants	Status	References
EA-230	Oligopeptide fragment from b-hCG	Immunomodulation	Intravenous	Sepsis	Phase 2	60	Completed	NCT02629874
hLF1-11	A synthetic cationic peptide; A peptide comprising the first eleven residues of hLF	Immunomodulation, direct antibacterial activity	Intravenous	Treatment of infectious complications among haematopoietic stem cell transplant (HSCT) recipients	Phase 2	8	Completed	NCT00509938
IMX-942	A synthetic cationic peptide; A derivative of IDR-1	Immunomodulation	Intravenous	Treatment of oral complication in patients being treated with chemotherapy	Phase 3	268	Completed	NCT03237325
AB103	A synthetic peptide mimetic of the T-lymphocyte receptor, CD28	Immunomodulation	Intravenous	Necrotizing soft tissue infections	Phase 3	290	Completed	NCT02469857
Talactoferrin	A recombinant form of the human glycoprotein, lactoferrin	Immunomodulation	Oral	Sepsis	Phase 2/3	1280	Suspended	NCT01273779
Omiganan	A synthetic cationic peptide; derivative of indolicidin	Immunomodulation	Topical	Topical antiseptic	Phase 3	30	Completed	NCT00608959
Brilacidin	A small molecule arylamide mimic of defensin	Direct antibacterial, potential immunomodulatory activities	Intravenous	Treatment of acute S. aureus skin and skin structure infections	Phase 2b	215	Completed	NCT02052388
rBPI21	Synthetic peptide	Direct antibacterial, potential immunomodulatory activities	Intravenous	Reduction of LPS-induced inflammatory sequelae, in particular aGvHD, in patients undergoing allogeneic HSCT	Phase 2	6	Terminated due to the lack of enrollment	NCT00454155

### M1 and M2 polarization of macrophage

Macrophages are professional phagocytes responsible for (1) preventing infections, (2) tissue repairing and (3) immunomodulation. Macrophage polarization, which refers to the process by which macrophages adopt distinct functional phenotypes in response to specific microenvironment signal or stimuli, is crucial for maintenance of homeostasis [124]. Macrophages can be classified into classically activated (M1) and alternatively activated (M2) macrophages, while M2 macrophages can be further categorized into M2a, M2b, M2c, and M2d subgroups [125]. These macrophages produce distinct functional phenotypes, for example, by having different secreted cytokines, cell surface markers and biological functions.

M1 macrophages are macrophages that possess pro-inflammatory phenotype [126]. Macrophages can be polarized into M1 macrophages in response to PAMPs (e.g. LPS from *E Coli*, PGN from *S aureus*) and Th1 cytokines (e.g. TNF-α and IFN-γ). They can be characterized by the expression of surface markers such as CD80, CD86, TLR-2, TLR-4, MHC-II and iNOS [127]. Upon polarizing into M1 phenotype, the M1 macrophages secrete various pro-inflammatory chemokines and cytokines (e.g. IL-6, IL-12, IL-1α, IL-1β, TNF-α), which further polarize more unpolarized macrophages into the M1 state [128]. Key transcription factors, such as NF-κB and STAT1, are also known as the major pathways responsible for the activation of M1 macrophages [129]. On the other hand, M2 macrophages are macrophages that produce anti-inflammatory responses, which protect the host from excessive inflammatory responses and tissue damage. M2 polarization occurs as a reaction to cytokines such as IL-4 and IL-13 [130]. Other cytokines such as IL-33 and IL-25 have also been shown to amplify M2 macrophage polarization [131]. M2 macrophages can be characterized by the presence of surface markers, such as CD163, CD206, CD209, and Ym1/2 [132]. Studies suggest that up-regulation of anti-inflammatory chemokines and cytokines, such as IL-10, CCL1, CCL17, CCL18, CCL22, CCL24 and TGF-β, can also drive the polarization of macrophages into M2 state [133, 134].

Activation of M1 polarization offers tremendous potential for efficient elimination of invading pathogens [135]. At the same time, switching macrophages from M1 to M2 state contributes to anti-inflammatory activity, tissue repair and wound healing [130]. Other than endogenous ligands, naturally occurring compounds such as diosgenin, celastrol and emodin, have also been shown to regulate macrophage polarization [136]. To date, the uses of TLR ligands and cytokines for M1/M2 macrophage polarization have been extensively investigated. For example, it has been found that zoledronic acid could promote TLR-4-mediated polarization of M1 macrophage [137], while curcumin could induce the secretion of IL-4/IL-13 [138] and induce M2 macrophage polarization [139].

## Types of nanocarriers and their potential applications

**Fig. 2:**
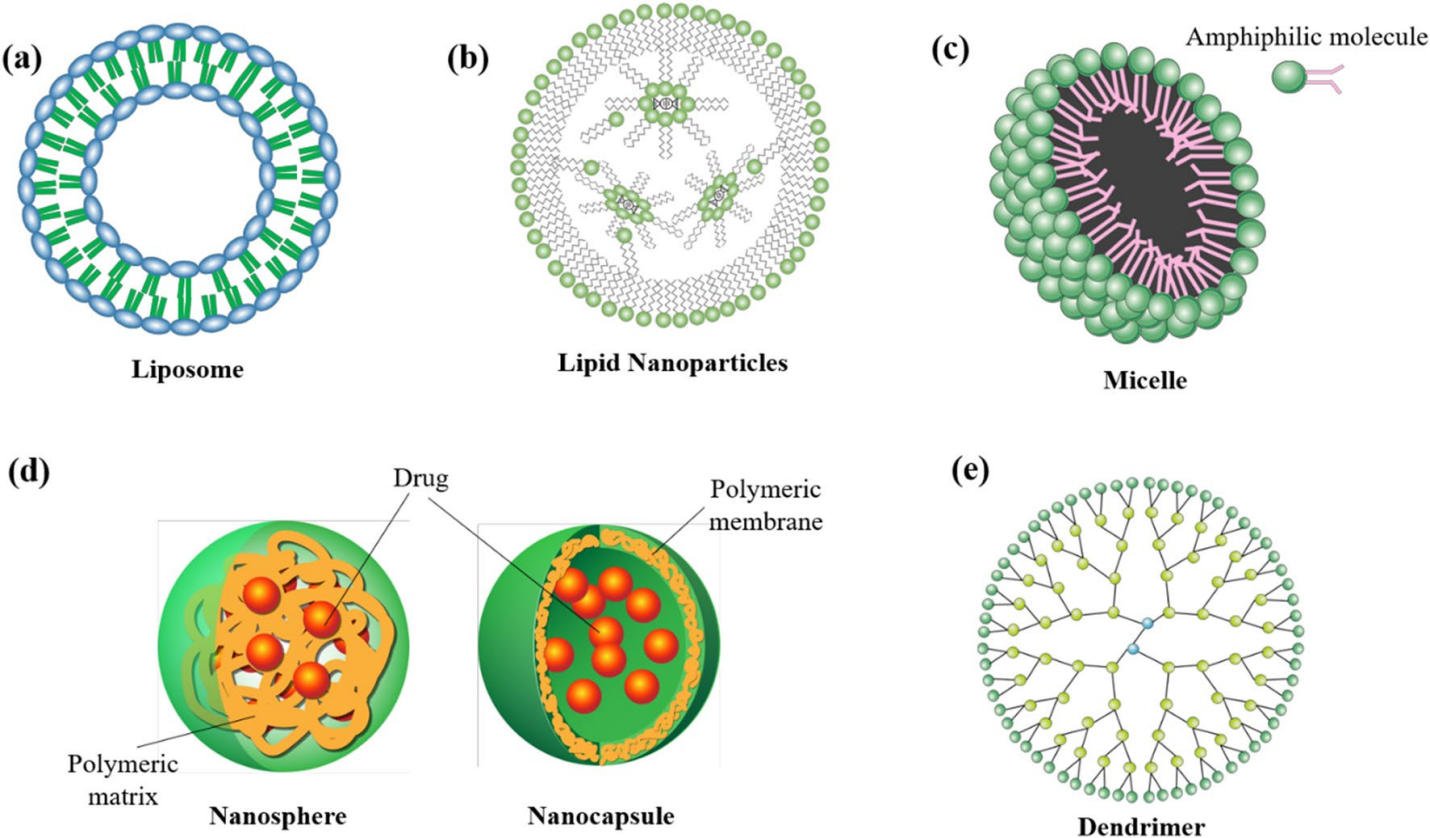
Structures of nanocarriers: **a** liposome, **b** lipid nanoparticles, **c** micelle, **d** polymeric nanoparticles (nanosphere and nanocapsule) and **e** dendrimer

Nanocarrier is a colloidal carrier system used for cargo transportation. Compared to conventional approaches, nanocarrier-assisted approaches readily improve the pharmacokinetics (e.g. drug absorption and circulation time) and pharmacodynamics (e.g. therapeutic index and specificity) of cargos. An ideal nanocarrier should be biocompatible, biodegradable, stable in body fluids, as well as tolerable for phagocytosis by immune cells. To date, numerous types of nanocarriers, such as liposomes, lipid nanoparticles, micelle, polymeric nanoparticles, metallic nanoparticles, and dendrimers, have been investigated extensively. Their designs and properties are summarized and compared in this section (Fig. 2).

### Liposome

Liposomes are self-assembling spherical vesicles that consist of at least one lipid bilayer. They are composed of either synthetic or natural phospholipids [140]. The characteristics of the constituent phospholipids, such as their lipid composition, size, surface charge and permeability, are key factors determining their physical and chemical properties. The loading of drugs into liposomes can be achieved by either passive or active methods [141]. For passive method, dried lipid is dissolved in an aqueous solution containing the load. Although this method is simple and easy to operate, only water soluble or hydrophilic drugs can be encapsulated, and their efficiency is relatively low. In contrast, active loading allows efficient drug loading with high intraliposomal drug concentration. For this method, drug is internalized due to the pH gradient across the environment outside and inside the liposome. The drug loading efficiency is linked to various factors, including the lipid composition of constituent phospholipids, aqueous solubility of the drug, and the nature of the transmembrane etc. [142].

Liposomes are available in various forms, known as conventional liposomes, PEGylated liposomes, and ligand-targeted liposomes [143]. The structures and compositions of conventional liposome is as shown in Fig. 2a. Compared to free drugs, the use of liposomal system can significantly enhance the specificity of drugs. Conventional liposomes are composed of either cationic, anionic, or neutral phospholipids, and cholesterol. In general, they consist of a lipid bilayer, which encloses an aqueous inner core formed by hydrophilic heads of phospholipids. The bilayer structure of liposomes allows encapsulation of hydrophilic drugs within the aqueous core, as well as entrapment of lipophilic drugs within the lipid portion of the bilayer membrane [144]. The unique ability of liposomal system for delivering both hydrophilic and lipophilic drugs, and their ability to prolong circulation time, reduce toxicity and improve biocompatibility of drugs makes them one of the ideal nanostructures for biomedical applications [145]. However, conventional liposomes are easily recognized by the mononuclear phagocyte system (MPS), and are rapidly cleared from the bloodstream [146]. The use of PEGylated liposome has therefore been introduced. The steric hindrance offered by PEG protects the liposome from phagocytosis. Studies have indicated that PEGylated liposomes have a substantially longer circulation time in bloodstream, with a reported half-live as high as 45 h in human [147]. However, while steric stabilization enables a longer circulation time, the enormous PEG coating may also hinder the biological interaction between liposome and its intended target, and thus lowering the drug efficacy. Ligand-targeted liposomes are modified liposomes coupling with ligands, such as peptides, proteins, aptamers and antibodies [148]. However, attaching ligands to liposomal surface may impart poor pharmacokinetics to liposome, limiting its therapeutic performance in vivo [149]. Therefore, newer generations of liposomes usually make use of a combination of the above designs to achieve specific targeting while improving pharmacokinetics. For example, it has been found that modification of ligand-integrating liposomes with PEG enables a better pharmacokinetics in vivo [150].

To date, numerous targeted-liposomal systems for the delivery of chemical drugs (e.g. cisplatin, oxaliplatin, lonidamine, indinavir, irinotecan), proteins/peptides (e.g. eptifibatide, growth factors such as VEGF, EGF, GDNF and VEGF), and nucleic acids (e.g. siRNA, plasmid DNA, miRNA) using targeting ligands such as RGD peptide, PTD (HIV) peptide, folic acid and transferrin, have been broadly used in the treatment of cancers, infectious diseases and neurodegenerative diseases [151]. Notably, some ligand-targeted liposomal systems, such as SGC-53 [152], anti-EGFR ILs-DOX [153], Lipovaxin-MM [154], MM-302 [155] and SGT-94 [156], have already completed clinical phase I/II trials in cancer patients. Most recently, the US FDA has approved Vyxeos, which is a liposomal system loaded with cytarabine and daunorubicin, for the treatment of acute myeloid leukemia. Furthermore, a PEGylated-magneto liposomal system loaded with multi-component drugs (i.e. drug abuse antagonist, latency reactivating agents and antiretrovirals) has also been reported [157]. This nano-formulation allows sustained drug release for up to 10 days with significant anti-HIV activity in primary CNS cells, offering potential for targeting viruses/bacteria that have migrated to the CNS. However, one should not confuse “liposome” with “polymersome”. Polymersome is a synthetic analogue to liposome that can be formed by the self-assembly of amphiphilic copolymers. Compared to liposomes, polymersomes are significantly more stable and can be engineered to allow bio-responsive drug delivery. However, owning to their stable and robust bilayer membrane, polymersomes usually have slow cargo release rate.

### Lipid nanoparticles

**Fig. 3:**
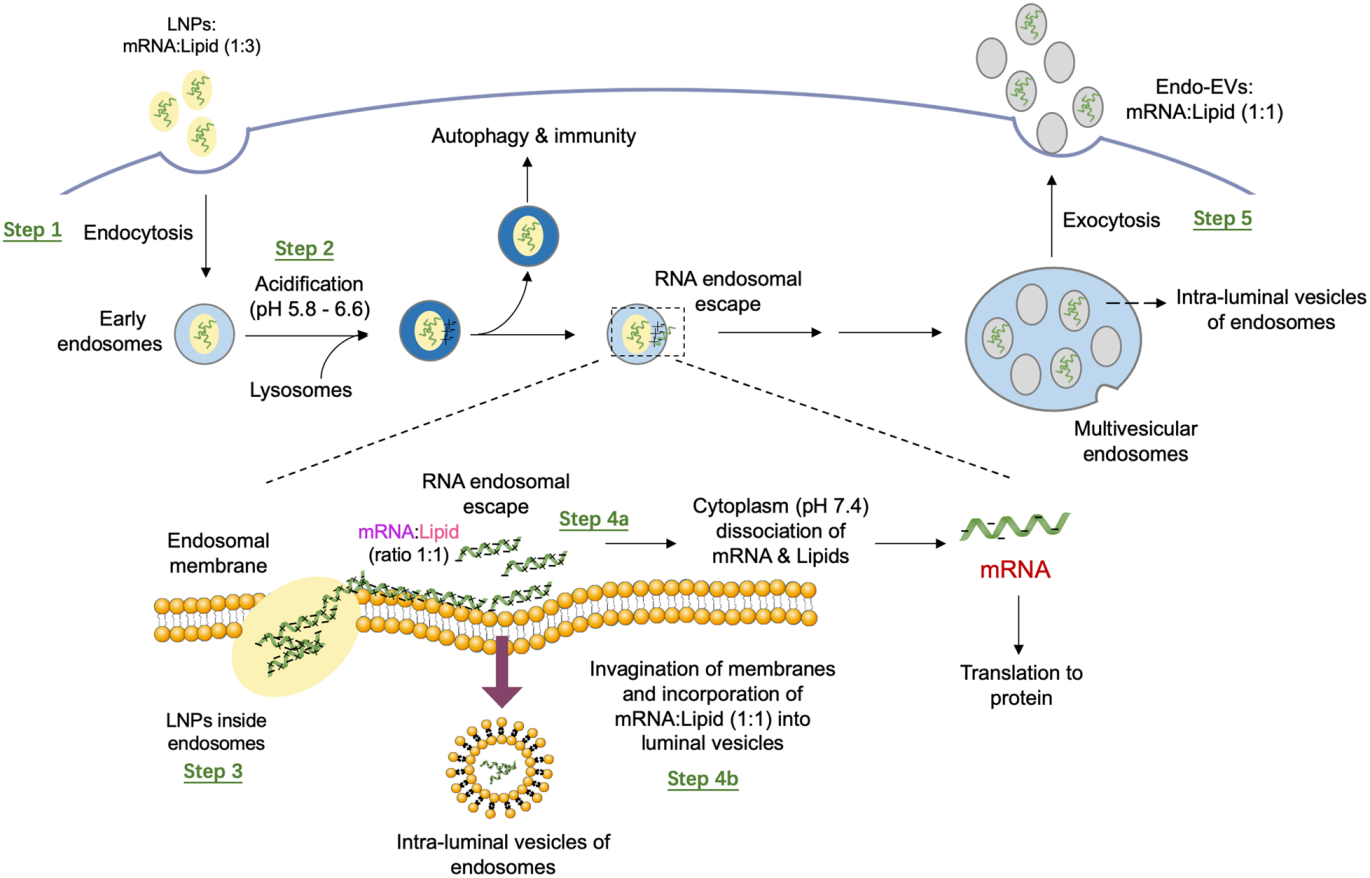
A mechanism describing the fate of LNP upon endocytosis. Reproduced with permission from reference [160]

Lipid nanoparticle (LNP) is a spherical viral-sized vesicle (80–200 nm) composed of ionizable lipids (Fig. 2b). Owning to their ionizability, LNPs are neutral at physiological pH and have a relatively low toxicity [158]. Upon endosome-engulfing, the acidic environment of endosome causes the LNP surface to become positively charged [159]. At low pH, the positively charged LNPs can complex with the negatively charged nucleic acids cargo (e.g. mRNA), forming a water-insoluble lipid-mRNA complex that has a neutral net charge. If such a lipid-mRNA complex is lipophilic, it can transport across the endosomal membrane during the fusion event, and dissociate in the cytoplasm, where the pH is neutral (~ pH = 7.6) [160]. This process eventually gives rise to the release of mRNA cargo intracellularly for protein synthesis (Fig. 3). LNPs usually contain cholesterol and a helper lipid to help with the filling of gap between lipids, and a PEG to reduce clearance by MPS [161]. The efficacy of LNPs can be substantially affected by the relative amount of lipid components, and therefore optimization of the amount of ionizable lipid, cholesterol, helper lipid, and PEG is critical for different applications [162]. Furthermore, the size, lipid type and surface charge of LNPs are also key parameters that affect their behavior in vivo. Liposomes and LNPs are similar by design, but they are slightly different in composition and functions. While liposomes are composed of cationic, anionic or zwitterionic lipids, LNPs are mainly composed of ionizable lipids or other lipid materials with cholesterol, neutral helper lipids and PEG-lipids. In contrast to conventional liposomes that have one or more rings of lipid bilayer surrounding an aqueous pocket, not all LNPs have such a contiguous bilayer. Some LNPs have a structure similar to micelles that encapsulate cargo in a non-aqueous core.

In application, LNPs can be used as delivery vehicle for both hydrophilic and lipophilic drugs. Notable examples include the lymphocyte-targeting LNPs recently developed by Kraft and the co-workers [163]. In this study, three antiretroviral drugs including lopinavir (hydrophobic), ritonavir (hydrophobic) and tenofovir (hydrophilic) were encapsulated in LNPs. This formulation enables a long-lasting drug profile in plasma and an improved drug levels in lymph nodes of macaques models. Furthermore, LNPs are especially designed for encapsulating nucleic acids (e.g. DNA, siRNA, mRNA and saRNA).

Non-viral vectors have emerged as a safer and versatile alternative to viral vectors (e.g. lentivirus and adenovirus vectors) due to their absence of immunogenic viral proteins. Among the non-viral gene vectors, LNPs have shown robust capability to deliver oligonucleotides in sizes ranging from several nucleotides (e.g. RNA) to millions of nucleotides (e.g. chromosome). The application of LNPs as delivery vehicle for RNA drugs was first approved by the US FDA in 2018 [164]. In late 2020, LNPs become widely known after some pharmaceutical industries developed COVID-19 vaccines using PEGylated LNPs as the delivery vehicle for the fragile mRNA strands [145]. Notable examples include the most advanced mRNA-based vaccines (e.g. mRNA-1237 and BNT162), developed by Moderna, Biontech, Pfizer and Fosun Pharma. The clinical trials investigating the safety, immunogenicity, tolerability and potential efficacy of these vaccine formulations are still ongoing (NCT04368728, NCT04380701, NCT04283461).

### Micelle

#### Phospholipid micelle

Phospholipid micelle is a colloidal suspension formed by self-assembling of surfactant phospholipid molecules [165]. It is structurally similar to cellular membrane which is also composed of phospholipids. This similarity allows micelles to be incorporated into cellular membrane or even travel across them. The hydrophilic shell and nanoscale dimension (usually have sizes ranging from 5 to 100 nm) of micelle not only lower the toxicity of the load, but also lower their recognition and rapid clearance by MPS. A typical micelle is formed in water whereby its hydrophilic region facing the outside surface, that is the aqueous environment, and the lipophilic tails sequester to form a lipophilic core (Fig. 2c). However, when formed in a lipophilic solution, for example fat, the structure of the micelle will be reversed. When compared to liposome that consists of a lipid bilayer and a hydrophilic core, a typical micelle has a lipophilic core and has been commonly used for the delivery of a board range of drugs that have poor solubility in water [166]. Furthermore, micelles have a smaller dimension and thus their drug-loading capacity are smaller when compared to liposomes [167, 168]. Phospholipid micelles are also known as sterically stabilized micelle when they are prepared using PEGylated lipid (e.g. DSPE-PEG_2000_). While small molecule drugs are encapsulated in the core, peptide drugs can be resided in the PEG polymer. Most recently, Esparza et al. reported a PEGylated phospholipid micelle for the delivery of an amphiphilic water-soluble peptide drug [169]. In this work, the PEGylated phospholipid (DSPE-PEG_2000_) self-assembles to form a sterically stabilized micelle. The amphiphilic peptides then self-associate with the micelle via molecular interaction and transition to an active alpha-helical conformation. Since the peptide is not covalently conjugated to the micelle, it can easily leave the micelle and bind to the receptor of interest.

#### Polymeric micelle

Micelles can also be formed by self-association of amphiphilic copolymers in aqueous solution. This type of micelles is known as polymeric micelle. They have a lipophilic core covered by a hydrophilic shell. In general, cargos can be loaded in polymeric micelles by chemical conjugation or physical entrapment. The rate of drug release depends on the way of loading. For instance, drugs that are chemically conjugated to polymeric micelles are released by surface erosion or degradation of the polymer, while physically entrapped drugs are released by diffusion [165]. Polymeric micelles that are ligand-conjugated can also achieve a higher efficacy and site-specificity, and thus achieving a better therapeutic outcome [170]. Polymeric micelles can be synthesized using many different polymers. The choice of polymer, however, must be biocompatible and biodegradable. For example, PEG is a common hydrophilic polymer used for micelle fabrication due to its water-soluble, non-toxic, and non-ionic nature [171].

To date, polymeric micelles have been used as versatile delivery carriers for various drugs and siRNAs [172]. Surface modification with various moieties (e.g. cell penetrating groups and/or stimuli-responsive groups) can impart additional functions to micelles, giving rise to multifunctional and “smart” polymeric micelles. Notable examples of stimuli-responsive polymeric micelles include (1) doxorubicin-loaded chondroitin sulfate-histamine micelle (pH-responsive) [173], (2) GFP siRNA-loaded Poly(D,L-lactic co-glycolic acid)/linear PEI micelle (redox-responsive) [174], (3) curcumin-loaded dialkoxycyanostilbene polymethacrylate-b-PEO micelle (UV light-responsive)[175], (4) paclitaxel/rifampicin-loaded PEO-b-P(LGA-co-COU) micelle (NIR light-responsive) [176], (5) doxorubicin-loaded pluronic F105 micelle (ultrasound-responsive) [177], and 6) doxorubicin/SPIO-loaded PEG-b-PCL micelle (magnetic field-responsive) [178]. Furthermore, the use of polymeric micelles for the delivery of siRNAs, such as MDR1 siRNA, Bcl-2siRNA, GFP/VEGF siRNA, and XIAP siRNA, have also been reported [172]. These “smart” polymeric micelles offer significant potential in the area of therapeutic delivery.

### Polymeric nanoparticles

Polymeric nanoparticles (PNPs) are colloidal particles made of biocompatible and biodegradable polymers. PNPs (Fig. 2d) usually have a size ranging from 1 to 1000 nm and can be synthesized in different morphological structures, known as nanocapsule and nanosphere [179]. Nanocapsule is a reservoir system that a drug is distributed within a cavity formed by the outer polymeric membrane. Nanosphere is a matrix system that a drug is embedded in the polymeric matrix. Both synthetic polymers (e.g. poly(lactide-*co*-glycolides) (PLGA), polylactides (PLA)) and natural polymers (e.g. chitosan, alginate, dextran and pectin), can be used for the synthesis of PNPs [180]. PNPs can be fabricated using solvent evaporation, emulsification/solvent diffusion, emulsification/reverse salting out and nanoprecipitation methods [181]. The composition of polymer, drug-to-polymer ratio, solubility of drug, and pH value are important parameters that affect the stability, release profile and particle size of PNPs [182].

To date, PNPs are widely used as nanocarriers for the delivery of a variety of drugs including small molecules, oligonucleotides (e.g. ssDNA, dsDNA, RNA), peptides, and proteins, which are either dissolved, encapsulated, entrapped within the polymeric core, or affixed to the polymeric surface. The use of PNPs system for drug delivery offers many advantages over the “free drug”, including (1) sustained/controlled drug release, (2) stable delivery of labile drugs such as protein drugs, and (3) allow surface modification for targeted drug delivery [183, 184]. PLGA and PLA are among the most commonly used synthetic polymers for biomedical applications. These polymers, upon hydrolysis, are degraded into their constituent monomers, glycolic and/or lactic acids, which are then excreted out of the body. Owning to their biocompatibility and biodegradability, many PLGA- and PLA-based drug delivery systems have already been approved by the US FDA. A review article reported by Wang and the co-workers summarizes a list of FDA-approved PLA/PLGA-based drug products [185]. For example, Lupron Depo, a PLGA/PLA-based medication, was first approved by the US FDA for the treatment of advanced prostate cancer in 1985.

Ever since their first approval, scientists have continued to bring forward new innovations in the design of PNPs-based products. For example, intravenous (IV) injection, nasal and oral administration of PLGA-based formulations for sustained release of antitubercular drugs, such as pyrazinamide, isoniazid and rifampicin, have been developed [186–188]. Following a single administration, a sustained level of the drug load could be detected in the circulation for up to 6–9 days. The absolute bioavailability of the drug was found to be > 10 folds higher than that of the free drugs. The use of natural PNPs, such as chitosan, for the delivery of antibiotics (e.g. levofloxacin and ceftazidime) have also been reported [189, 190]. The developed formulations were found to be safe and non-irritant for topical ophthalmic use. Compared to free drug solution, chitosan NPs could effectively improve the retention time of drug in eyes and enhance their antibacterial activities. More recently, studies have also focused on the development of PNPs-based formulations for the delivery of potential therapeutic agents against COVID-19 [181, 191]. For example, the self-injectable remdesivir-loaded PLGA nanoparticles, namely SelfExRem, has been developed by Patki and the co-workers. Upon injected into an aqueous environment, SelfExRem undergoes a rapid solution-soft gel transition, which allows a controlled release of remdesivir at a steady rate for 2 days. Furthermore, PNPs have been used as nanocarriers for the delivery of immunomodulatory drugs (e.g. small molecules, siRNA, peptides, proteins) [192]. Their applications in the management of infectious diseases are discussed in the "Nanocarriers for delivery of immunomodulatory drugs and vaccine adjuvants" section.

### Metallic nanoparticles

Metallic nanoparticles (MNPs) are nanoparticles derived from metals with dimension ranging from 1–100 nm [193]. It has been reported that nanoparticles are preferably accumulate in spleen, liver and lymph nodes, and may persist in biological system for prolonged period [194]. The chemical inertness of noble metals such as silver and gold renders them the ideal raw materials for the formation of MNPs. The large surface charge and surface area of MNPs also enable them to interact with the surface of bacterial membranes electrostatically, interrupt their integrity and thereby killing them. For example, silver nanoparticles (AgNPs) are known to have remarkable antibacterial efficacy against a range of bacteria such as *Bacillus subtilis, Pseudomonas aeruginosa* and *Staphylococcus aureus* [195]. The antimicrobial activity of AgNPs are attributed to their ability to perturb bacterial cell wall and promote lysis of bacterial membrane [196]. The combination of MNPs and drug molecules, for example by adsorbing or coupling drugs onto the surface of MNPs, are found to have synergistic effect on combating bacterial infections [197]. Over the past decades, the use of MNPs as nanocarriers for drug molecules have been extensively studied. For example, Pal et al. developed an AMP-conjugated AgNPs, which combines the potency of AMPs and AgNPs, and found that the conjugate had enhanced biological activities against *E. coli*[198]. On the other hand, Moyano et al. reported the use of AuNPs for modulating the host immune responses [199]. The AuNPs with hydrophobic functionalization (e.g. TEGOH and ZDiPen) successfully decreased the production of cytokines e.g. TNF-α, whereas the AuNPs with hydrophilic functionalization (ZDiMe) did not affect the secretion of TNF-α.

In addition to AuNPs and AgNPs, magnetic iron oxide nanoparticles (MIONPs) have also attracted considerable attention due to their superparamagnetism, ease of separation and large surface area. To date, various chemical and physical methods, such as liquid phase methods, microemulsion, gas/aerosol phase methods, sol–gel methods, polyols methods, and hydrothermal reaction methods, have been used for the synthesis of MIONPs [200]. However, owning to the strong attraction force among particles, MIONPs that have bare surface tend to agglomerate. Coating MIONPs with inorganic or organic molecules, such as drugs, oligonucleotides, polymers (e.g. PEG, Poly(d, l-lactide), chitosan, dextran), polypeptides and surfactants, can make them functionalized and/or biocompatible to the biological environment [201]. In a recent study, Cobaleda-Siles et al. developed a MIONPs-based nanocarrier and investigated its ability for the delivery of Poly(I:C) (a TLR3 agonist) [202]. The nanocarrier is capable of trafficking to the lymph nodes and the endosomal compartments, and is able to activate the TRIF-mediated TLR3 signaling pathway. Furthermore, Ane et al. developed a lipid-coated ^67^Ga-MIONPs and found that the nanoparticles can be used for image-tracked delivery of antigen and CpG (a TLR9 agonist) to the lymph nodes [203]. This nanoparticle is easy to assemble, biocompatible, and can efficiently monitor the delivery in vivo.

### Dendrimer

Dendrimer is a regularly branched, highly ordered three-dimensional polymeric molecule composed of branching groups covalently attached to a core (Fig. 2e) [204]. They can be synthesized by click chemistry, divergent or convergent methods. Their structures are influenced by various factors such as surface modification, pH value, temperature, ionic strength and spacer length. The lipophilic cavity and hydrophilic surface groups of dendrimers allow them to carry drugs that have poor water solubility. Owning to their uniform architecture, the applicability of dendrimers has also been extended to controlled/sustained drug release [205]. Furthermore, the polyvalency surfaces of dendrimers enable the formation of drug- or ligand-conjugated dendrimers [206]. For example, mannose-modified dendrimer can enter macrophage via CD206 (mannose receptor)-mediated endocytosis [207]. Gajbhiye et al. reported a mannose-functionalized poly(propyleneimine) dendrimer and found that the dendrimer could significantly enhance the cellular uptake of zidovudine by macrophage and lower the cytotoxicity of the load [208].

To date, dendrimers are being extensively investigated as nanocarriers in many biomedical applications [209]. The use of non-modified dendrimers as nanocarriers for the delivery of cancer drugs, including capecitabine, cisplatin, doxorubicin, paclitaxel, and methotrexate, have been reported. For example, encapsulating capecitabine in a G4/PAMMA complex could significantly reduce its side effects and inhibit the growth of tumor in a colorectal mouse model [210]. The drug loading capacity of dendrimers are highly dependent on their generation, whereas high-generation dendrimers always have toxicity issues. Shielding the positive charge of dendrimer surface is an effective strategy to reduce its toxicity. It can be easily achieved by conjugating the primary amines on the dendrimer surface with shielding groups such as PEG chains, alkyl chains and hydroxyl chains [211–213]. However, this method does not allow sustained drug release due to the open structure of dendrimers. Making use of the surface functional groups of dendrimers, some research groups reported the use of covalent-drug dendrimer conjugates. For example, Lee et al. reported a PEGylated dendrimer-doxorubicin conjugate and found that the conjugate had an improved delivery efficiency to tumor [214]. The covalent-drug conjugate also improves the drug solubility and allows a longer circulation time. In recent years, dendrimers responsive to stimulus such as pH, redox, enzymes and temperature changes, have been developed. For example, Khandare et al. designed a paclitaxel-conjugated hydroxyl-shielded dendrimer and used it for esterase-dependent delivery of paclitaxel to A2780 human ovarian cancer cells [215]. Notably, this dendrimer enabled a 10-fold higher cytotoxicity compared to the free drug.

## Nanocarriers for delivery of immunomodulatory drugs and vaccine adjuvants

Immunomodulators targeting PRRs may be beneficial to treat infectious diseases and their associated complications. However, their therapeutic performances can be negatively affected by (1) high immune-mediated toxicity, (2) poor solubility and (3) bioactivity loss after long circulation. Recently, nanocarriers have emerged as a very promising tool to overcome these obstacles owning to their unique properties such as sustained circulation, desired bio-distribution, and preferred pharmacokinetic and pharmacodynamic profiles [216–219]. In general, nano delivery can be classified into passive and active approaches [220]. In passive targeting, the cargo is stabilized in a nanocarrier to avoid premature degradation in the biological environment. This can be achieved by covering the nanocarrier by a distinct type of hydrophilic materials e.g. polyethylene glycol (PEG). The hydrophilic surface of the nanocarrier protects the cargo against phagocytosis and improve their overall circulation time. In active delivery, nanocarriers are conjugated with ligands such as peptide, aptamer, antibody, glycoprotein, or polysaccharide to enhance specificity and facilitate internalization into target cells [221]. In this section, representative examples of nanocarrier-assisted immunomodulator delivery, and their applications in managing infectious diseases are discussed (Table 3).

**Table 3:** A summary of nanocarrier-assisted delivery of immunomodulators for potential management of infectious diseases and the associated complications

Nanocarrier	Target site / Type	Load	Formulation	Model	Role	Potential Application	Refs.
Liposome	TLR7/8	Resiquimod	Hydrogenated (soy) L-a-phosphatidylcholine, 1,2-distearoyl-sn-glycero-3-phospho-(1′-rac-glycerol) (DOPG), cholesterol and D-a-tocopherol	Murine	Immunomodulatory drug	Visceral *L. donovani* infection	[224]
	TLR3, TLR9	Poly I:C and noncoding plasmid DNA (pDNA)	Cationic liposome; 1,2-dioleoyl-3-trimethylammonium propane	Kitten	Immunomodulatory drug	Feline herpesvirus-1 infection	[225]
	TLR3, TLR9	Poly I:C and pDNA	LTC; Cationic lipid 1,2-dioleoyl- 3-trimentylammonium-propane (DOTAP) and cholesterol	Cat	Immunomodulatory drug	Bacterial respiratory tract infections in cat	[227]
	TLR3, TLR9	Poly I:C and pDNA	LTC; cationic lipid DOTAP and cholesterol	Dog	Immunomodulatory drug	Viral and bacterial infections in dog	[228]
	TLR1/2, TLR9	Pam3CysSK4 (PMA) and CpGs	DOTAP and cholesterol	Mice	Vaccine adjuvant	/	[232]
	STING	cdGMP	PEGlyated liposome; 1,2-dioleoyl-sn-glycero-3-phosphocholine (DOPC), DOPG, 1,2-dimyristoyl-sn-glycero-3-phosphocholine (DMPC), DSPE-PEG	Mice	Vaccine adjuvant	HIV-1	[271]
	STING	cGAMP	Pulmonary surfactant-biomimetic liposomes; 1,2-dipalmitoyl-sn-glycero-3- phosphocholine (DPPC), 1,2-dipalmitoyl-sn- glycero-3-phospho-(1'-rac-glycerol) (DPPG), DPPE-PEG, cholesterol	Mice and ferrets	Vaccine adjuvant	H1N1	[244]
	IDR	Nisin Z	Phosphatidylcholine (PC), PC-cholesterol, and PC–phosphatidylglycerol (PG)–cholesterol	In vitro	Immunomodulatory drug	*Listeria monocytogenes*	[247]
	IDR	Polymyxin B	DPPC and cholesterol or 1-palmitoyl-2-oleoyl-*sn*-glycero-3-phosphocholine (POPC) and cholesterol	In vitro	Immunomodulatory drug	Gram negative bacterial strains	[249]
	IDR	LL-37	1,2-distearoyl-sn-glycero-3-phosphocholine (DSPC), DSPE-mPEG_2000_ and cholesterol	3D epidermis model	Immunomodulatory drug	HSV	[250]
Polymersome	TLR8	CL075 (a synthetic imidazoquinoline)	Poly (ethylene glycol)-bl-poly(propylene sulfide) (PEG-bl-PPS)	Neonatal mice	Vaccine adjuvant	Vaccine delivery system for human neonatal vaccines against infections e.g. *Mycobacterium tuberculosis*	[236]
Micelle	STING	cGAMP	PEG-b-PR copolymer	Clinical	Vaccine adjuvant	Avian and swine influenza	[242]
	IDR	DP7-C	A modified DP7 (an active IDR) with cholesterol to form an amphiphilic conjugate	Zebrafish and mice	Immunomodulatory drug	Bacterial infection	[252]
Lipid nanoparticle	TLR7	mRNA	An ionizable lipid, a phospholipid, a sterol, and a lipid-anchored PEG	Clinical	Vaccine adjuvant	SARS-CoV-2	[235]
Polymeric nanoparticle	TLR4; TLR1/2; TLR9; NOD2	LPS; PAM; CpG DNA, MDP	N-trimethyl chitosan (TMC) nanoparticles	Mice	Vaccine adjuvant	/	[233]
	TLR, macrophage polarization	siERN1	Nanoparticles synthesized using folic acid, PEG, PEI, PBAA, a cell-penetrating peptide	Mice	Immunomodulatory drug	Collagen-induced arthritis and LPS-induced over-inflammation	[230]
	TLR	/	PLGA, PLA nanoparticles	Mice	Inherent immunomodulatory effect of nanoparticles	Sepsis	[231]
	TLR4, TLR7	MPL, R837	PLGA nanoparticles	Mice, rhesus macaque	Vaccine adjuvant	H1N1	[234]
	NOD1, NOD2	CL235 [tetradecanoyl-δ-D-glutamyl-L)-meso- lanthionyl-(D)-alanine]…, CL365 [6-O-stearoyl-N-glycolyl-Murabutide]…	Poly(Lactic Acid) (PLA) nanoparticle coating with HIV-1 Gag p24 antigen	Mice	Vaccine adjuvant	Safe and efficient vaccine for infectious diseases	[237]
	NOD1	iE-DAP	Poly (3-hydroxybutyrate-co-3-hydroxyvalerate) (PHBV) nanoparticle	In vitro	Vaccine adjuvant	Potential for being used in the defense against infections	[238]
	NOD2 and TLR4 agonists	monophosphoryl lipid A, muramyl dipeptide	PLGA nanoparticle	Mice	Vaccine adjuvant	Influenza infection	[239]
	Immunostimulation of macrophage	Amphotericin B	PLGA nanoparticle	In vitro	Immunomodulatory drug	Visceral leishmaniasis	[258]
	Immunostimulation of macrophage	Rifampicin	PLGA nanoparticle	In vitro	Immunomodulatory drug	anti-tubercular	[259]
	Immunostimulation of macrophage	Curcumin	Mannosylated chitosan nanoparticle	Rat	Immunomodulatory drug	Visceral leishmaniasis	[262]
	Immunostimulation of macrophage	AHP	PEI-modified PLGA nanoparticles	Mice	Immunomodulatory drug	H5N1	[268]
	Macrophage polarization	ITA	Polyester polymer-ITA biomimetic material	Mice	Immunomodulatory drug	Resolve infection-associated over-inflammation	[269]
Metallic nanoparticles	IDR	Esculentin-1a(1–21)NH_2_	AuNPs	In vitro	Immunomodulatory drug	*P. aeruginosa*	[253]
	IDR	Cecropin-melittin	AuNPs	Mice	Immunomodulatory drug	Sepsis	[254]
	IDR	Nisin	AuNPs	In vitro	Immunomodulatory drug	Multi-drug resistance and non-multi-drug resistant *Staphylococcus aureus* and *Enterococcus faecalis*	[255]
Dendrimer	TLR4	Glucosamine	Polypropyletherimine dendrimer	Rhesus macaques	Immunomodulatory drug	Cytokine storm in severe bacterial diarrhea	[229]

### TLR signaling

Targeting TLR has been a promising strategy for modulating the host’s immune response. Leishmaniasis is a zoonotic disease caused by the causative agent Leishmania. Depending on the species, the disease can progress into cutaneous (e.g. *L. Mexicana*, *L. tropica* and *L. major*), mucosal (*L. braziliensis*) or visceral (*L. infantum* and *L. donovani*) leishmaniasis. In particular, *L. donovani* is resistant to macrophage’s phagolysosome, and therefore can proliferate and spread through the circulation system. If left untreated, visceral leishmaniasis can result in death. Resiquimod, which is a FDA-approved TLR7/8 agonist, has demonstrated potency for the treatment of cutaneous Leishmania infection [222]. However, the lipophilic nature of resiquimod has limited its parental use in vivo. Prior research has shown that encapsulation of resiquimod in polymer microparticles could significantly enhance the host immune response [223]. In a later study, Peine et al. encapsulated resiquimod in liposome and investigated its efficacy against visceral *L. donovani* infection in murine models. The liposomal system successfully increased the production of IL-10 cytokines and significantly decreased the parasitic load in bone marrow, spleen and liver [224]. Furthermore, a study conducted by Contreras et al. shows that the administration of liposome-toll like receptor complex (LTC) into both oropharynx and nares of kitten could significantly decrease the amount of feline herpesvirus-1 (FHV-1) DNA [225]. Another study by Wheat et al. demonstrated a dose-dependent upregulation of innate immunity in the nasopharynx upon intranasal administration of LTC [226]. Most recently, Wheat and the co-workers proposed a modified cationic LTC formulated with poly I:C (a TLR3 agonist) and noncoding plasmid DNA (a TLR9 agonist). Upon mucosal administration, the modified TLC upregulated the production of pro-inflammatory cytokines and induced innate immune response in cats effectively [227]. Interestingly, the same research group also demonstrated that the mucosal administration of TLR3- and TLR9-complexed TLC could potently activate the local innate immunity in dogs without affecting the composition of local microbiome [228].

Apart from infectious diseases, the uses of TLR antagonists for the treatment of infection-associated inflammation were also reported. For example, Islam et al. proposed a polypropyletherimine dendrimer glucosamine (a TLR4 antagonist) and found that the dendrimer was able to resolve the “cytokine storm” condition. Oral administration of this dendrimer significantly lowered the level of pro-inflammatory cytokines in rhesus macaques, demonstrating the potential of nano-assisted TLR4 antagonist for controlled resolution of infection-associated inflammation [229]. Other examples also include the macrophage targeting nanomedicine FA − PEG − R − NPs@siERN1 developed by Feng and the co-workers [230]. The nanoprodrug allows sustained release of siERN1, which is an inhibitor of the MyD88-dependent TLR signaling pathway. The result shows that siERN1 could downregulate the expression of the MyD88-dependent TLR pathway molecules such as IRAK4, p-p38/p38, p-JNK/JNK and p-NF-κB/NF-κB. In another study, Casey et al. developed a cargo-less PLGA-PLA nanoparticles that exhibit charge-dependent inherent immunomodulatory properties[231]. Interestingly, the nanoparticles prepared using anionic surfactant (ethylene-alt-maleic acid) (PEMA-NPs) display broad inhibitory activity to both intra- and extracellular TLR ligands, whereas the nanoparticles prepared using neutral surfactant poly(vinyl alcohol) (PVA-NPs) only marginally inhibit the secretion of pro-inflammatory cytokines. The administration of PEMA-NPs prior to or following lethal challenges significantly enhanced the survival rate of LPS-induced sepsis mouse model.

Ongoing variations in major antigenic targets can limit the efficacy of vaccination. Adjuvants can readily improve the immunogenicity of vaccines and are important for optimal antigen-specific immune responses. Bal et al. reported that co-encapsulation of antigens and TLR ligands (i.e. Pam3CysSK4 (PMA) or CpGs) in a dioleoyl-3-trimethyl ammonium propane (DOTAP) liposomal system could activate the innate immune response and improve the immunogenicity [232]. The same research group proposed the use of N-trimethyl chitosan (TMC) polymeric nanoparticles for encapsulating immunomodulators such as TLR agonist LPS, PAM, CpGs, and found that co-encapsulation of an immunomodulator with the antigen into TMC could significantly enhance the immunogenicity of the vaccine [233]. Interestingly, co-delivery of TLR4 and TLR7 adjuvants using PLGA nanoparticles was found to induce synergistic production of cytokines, resulting in enhanced T cells responses and increased antigen-specific antibodies in comparison to immunization with a single TLR ligand [234].

Intriguingly, numerous SARS-CoV-2 vaccines have recently been approved for emergency use. The approved vaccines for SARS-CoV-2 developed by Moderna and Pfizer make use of LNPs as the delivery vehicle. At the cellular level, the mRNA-loaded LNP enters dendritic cells and produce a high level of SARS-CoV-2 spike protein in cells. The LNP not only offers protection to the mRNA load by specifically delivering it to lymphatics, but also facilitates the translocation of protein in the lymph nodes. In addition, the ssRNA and dsRNA delivered in the mRNA vaccines bind to the TLR in the endosome and activate the innate immune responses via the production of IFN-I and other multiple inflammatory mediators, which provide necessary signals for triggering a stronger adaptive immune responses [235]. On the other hand, human infants are more susceptible to infectious diseases due to their early-life immunity system. Their dendritic cells have particularly low production of IL-17p70 and impaired T_H_1 responses, which are especially important for vaccination-induced protection. Although imidazoquinoline (TLR8 agonist) could robustly activate newborn dendritic cells, they may lead to reactogenicity when delivered in soluble form. Recently, Dowling et al. constructed a CL075 (a synthetic imidazoquinoline)-loaded polymersome (CL075-PS) that can be selectively uptaken by dendritic cells [236]. Compared to whole bacterial vaccine BCG, the TLR8 agonist adjuvanted formulation (CL075:Ag85Bp25-PS) not only induced a comparable maturation profile of newborn dendritic cells, but also induced a stronger production of IL-12p70.

### NOD signaling

NOD1 and NOD2 are intra-cytoplasmic sensors for pathogens. γ-D-Glu-mDAP, also known as iE-DAP, is a dipeptide that can be found in the PGN of all Gram-negative bacteria and certain types of Gram-positive bacteria. Recognition of iE-DAP by NOD1 activates the NF-κB signaling pathway and induces the production of inflammatory cytokines. On the other hand, MDP, a NOD2 agonist, is the minimal bioactive peptidoglycan motif that can be found in almost all kinds of bacteria. Pavot and co-workers designed a NOD1-and NOD2 ligands-encapsulated poly(Lactic Acid) (PLA) nanoparticle coated with HIV-1 Gag p24 antigen on the surface. The encapsulated ligands were effectively internalized by dendritic cells and enabled a higher activation of the NF-κB pathway [237].

Further studies also indicate that intradermal and nasal administration of MDP-encapsulated N-trimethyl chitosan nanoparticles could increase the immunogenicity of antigens [233]. In a separate study, Mauricio et al. developed an iE-DAP-loaded biocompatible poly (3-hydroxybutyrate-co-3-hydroxyvalerate) (PHBV) nanoparticle, and found that the nanoparticle could be taken up by cells effectively [238]. Intriguingly, this nanoparticle enables a sustained and controlled release of cargo. The encapsulated iE-DAP was able to induce the production of a higher level of pro-inflammatory IL-6 and TNF-a when compared to its free form []. Interestingly, co-encapsulation of both NOD2 and TLR4 agonists in PLGA nanoparticles has also been reported. In vitro data shows that the combined stimulation of NOD2 and TLR4 receptors allow synergistic activation of the AP-1/NF-κB-dependent transcription. The complex adjuvant was found to initiate the pro-inflammatory innate immune responses by enhancing the cytokine production in vitro and in vivo, and was able to protect the mice model against influenza infection [239]. This study demonstrates that complex adjuvants activating multiple PRRs may be a promising alternative to individual adjuvant, and could potentially narrow the gap between whole pathogen vaccines and subunit vaccines.

### STING signaling

STING is an important mediator of innate immune response. Although free STING agonist e.g. cGMAP and their analogues can be taken up by some cells, the presence of extracellular cGAMP-cleaving enzymes and the inherent negative charge of cGAMP negatively affected the delivery of STING agonists in vivo [240, 241]. In this section, we summarize examples of nanocarrier-assisted delivery of STING agonists and their applications in vaccine immunogenicity.

Human immunodeficiency virus (HIV) is a virus that attacks cells in the immune system. If treated improperly, HIV can lead to acquired immunodeficiency syndrome (AIDS), which is a potential life-threatening chronic condition. Recently, several nanovaccine platforms for HIV have been proposed. For example, Aroh et al. prepared an ultra pH sensitive cGAMP-loaded micelle (PC7A) as an adjuvant for modulating the innate immune response [242]. The formulation was able to inhibit replication of two HIV-1 strains, IIIB and LAI, in peripheral blood mononuclear cells (PBMCs). Importantly, the authors compared the results using other adjuvants such as poly(I:C) (TLR3 agonist), R848 (TLR7/8 agonist), and CpG (TLR9 agonist). Although most of these agonists were able to induce inflammatory responses in PBMCs, none of them showed benefits in the inhibition of HIV-BaL in PBMCs, suggesting the potential of using cGAMP nanosystem as the adjuvant for HIV vaccine. In another study, Hanson et al. encapsulated a chemically synthesized cyclic di-GMP (cdGMP) in a PEGylated liposomal system that possess HIV gp41 peptide antigens [243]. Intriguingly, the liposomal system was able to redirect the cargo to the draining lymph nodes and block the systemic dissemination of free cdGMP in murine models, thereby avoiding systemic inflammation caused by the rapid distribution of cdGMP. The liposomal system also mediated the induction of type I IFN signaling in the draining lymph nodes and was found to elicit 3–4 folds higher activation of dendritic cells and macrophages compared to the unformulated cdGMP. Furthermore, nanovaccine platforms for other viruses e.g. influenza virus have also been developed. For example, Wang et al. developed a cGAMP-encapsulated pulmonary surfactant-biomimetic liposomal system (PS-cGMAP) and investigated its ability to induce activation of innate immunity [244]. The result indicates that PS-cGMAP robustly and rapidly, though only transiently, triggered the innate immune response. The expression of key mediators of inflammation such as Gmcsf, Tnf, Ifnb1 peaked at 12 h after administration of PS-cGAMP and resolved within 2 days. The transient activation of innate immune response was also shown to be sufficient to augment the subsequent cellular and hormonal adaptive immune responses.

### IDR peptides

IDR peptides (also known as AMP) are a class of short peptide that displays significant role in innate immunity [245]. They either act indirectly by modulating the host immune system, or directly by destroying the bacteria. In this section, only IDR peptides that possess immunomodulatory effects are discussed. Nisin Z is a bacterial antibiotic peptide that possess a similar mechanism as HDP. It has been reported that nisin Z engages in multiple signaling pathways, and is able to induce the secretion of chemokines (e.g. IL-8 and MCP-1) in human PBMC [246]. Benech et al. reported a nisin Z-encapsulating liposomal system and found that the system could effectively enhance the efficacy of nisin Z against *Listeria monocytogenes* [247]. Polymyxin B is another class of IDR peptide capable of activating the primary effector cells of the innate immune system [248]. Alipour et al. reported a polymyxin B-encapsulated liposomal system, and found that the encapsulation had a much lower minimum inhibitory concentration (MIC) against Gram negative bacterial strains compared to the free drug [249]. Another study conducted by Ron-Doitch et al. shows that encapsulation of LL-37, which is a cathelicidin peptide that plays a critical role in innate immunity, in a liposomal system improved the cellular uptake efficiency of LL-37 and lowered the cytotoxicity [250, 251].

Furthermore, studies relating to the self-assembly of IDR peptides against bacterial infections have also been reported. For example, Zhang et al. developed an amphiphilic conjugate DP7-C by adding cholesterol to DP7, which is a highly potent IDR peptide. Interestingly, DP7-C self-assembles in aqueous solution to form micelles that possess immunomodulatory effect on immune cells [252]. The DP7-C micelles could up-regulate innate immunity by increasing the production of cytokines. Indeed, DP7-C was also found to counterbalance the innate immune response stimulated by LPS [252].

Importantly, the synergy of conjugating IDR peptides to the surface of MNPs has also been reported. For example, Casciaro et al. evaluated the antibacterial activities of a esculentin-1a(1–21)NH_2_ (an IDR peptide)-coated AuNPs and found that the IDR peptide-coated AuNPs had a higher potency against *P. aeruginosa* compared to the free IDR [253]. In a separate study, Rai et al. conjugated cecropin-melittin (an IDR peptide that possess immunomodulatory activities) to AuNPs and found that the conjugate gave a lower minimum inhibitory concentration (MIC) compared to the free AuNPs [254]. Pradeepa et al. developed a nisin-functionalized AuNPs and found that the conjugate had an 8- to 32-folds lower MIC against both multi-drug resistant and non-multi-drug resistant *Staphylococcus aureus* and *Enterococcus faecalis* than its free form [255]. Importantly, the nisin-AuNPs displayed a minimal toxicity with low hemolycic activity.

### Targeting macrophage

Macrophages can be polarized into either M1 or M2 states in response to the surrounding environment, for example the presence of PAMPs or some cytokines. Targeting macrophages can be classified into passive and active approaches. In general, size and surface of delivery vehicles are responsible for passive targeting, while surface functionalization with ligand(s) attaching to the surface governs active targeting. Macrophages can be activated by different stimuli, such as polysaccharide and protein. M1 macrophages produce cytokines such as IL-12, IL-1β, IFN-γ, TNF-α, and co-stimulatory molecule CD80, which are important compounds to defense against invading pathogens [256].

Studies have shown that nanoparticles conjugated with mannosylate, such as mannan, mannose, or mannosamine, can modulate polarization of macrophages from M2 to M1 state [257]. For example, Barros et al. proposed a nanoscale drug carrier poly(D,L-lactide-coglycolide) polymeric nanosphere for the delivery of amphotericin B, which is an antifungal drug. In vitro result shows that this functionalized nanosphere was able to activate macrophage and produce pro-inflammatory cytokines such as IL-6 and TNF-α, with mannan-functionalized nanosphere being able to elicit the greatest immune response. The mannan-functionalized nanosphere was able to improve the efficacy of drug (Amphotericin B) against *L. infantum* by over 70 folds. The result also highlights the ability of this functionalized nanosphere in reducing the cytotoxicity of the loaded drug [258]. In a later study, Tukulula et al. proposed the use of curdlan-functionalized PLGA nanoparticle as a nanocarrier that possess immunostimulatory effect on macrophages. Curdlan is a known immune stimulating polymer. This nanocarrier allows sustained release of rifampicin. The nanocarrier was able to stimulate the generation of phosphorylated ERK (p-ERK) in macrophage, which is an upstream mediator of ROS and RNS [259]. Targeting macrophages can also be accomplished by galactose- and di-mannose, which are two conserved carbohydrates present on the surface of respiratory pathogens. Chavez-Santoscoy et al. developed a functionalized polyanhydride nanoparticles and found that the nanoparticles initiated the secretion of pro-inflammatory cytokines such as IL-6, IL-1b and TNF-α in alveolar macrophages [260]. Recently, Truong et al. developed a high-throughput microfluidic method for the generation of cargo-less immunomodulatory polymeric nanoparticles [261]. In this study, a set of 12 nanoparticles were developed and their ability to modulate the LPS-induced macrophage responses were investigated. The developed nanoparticles demonstrated board inhibitory effect against pro-inflammatory cytokines e.g. IL-6 and TNF-α.

**Fig. 4:**
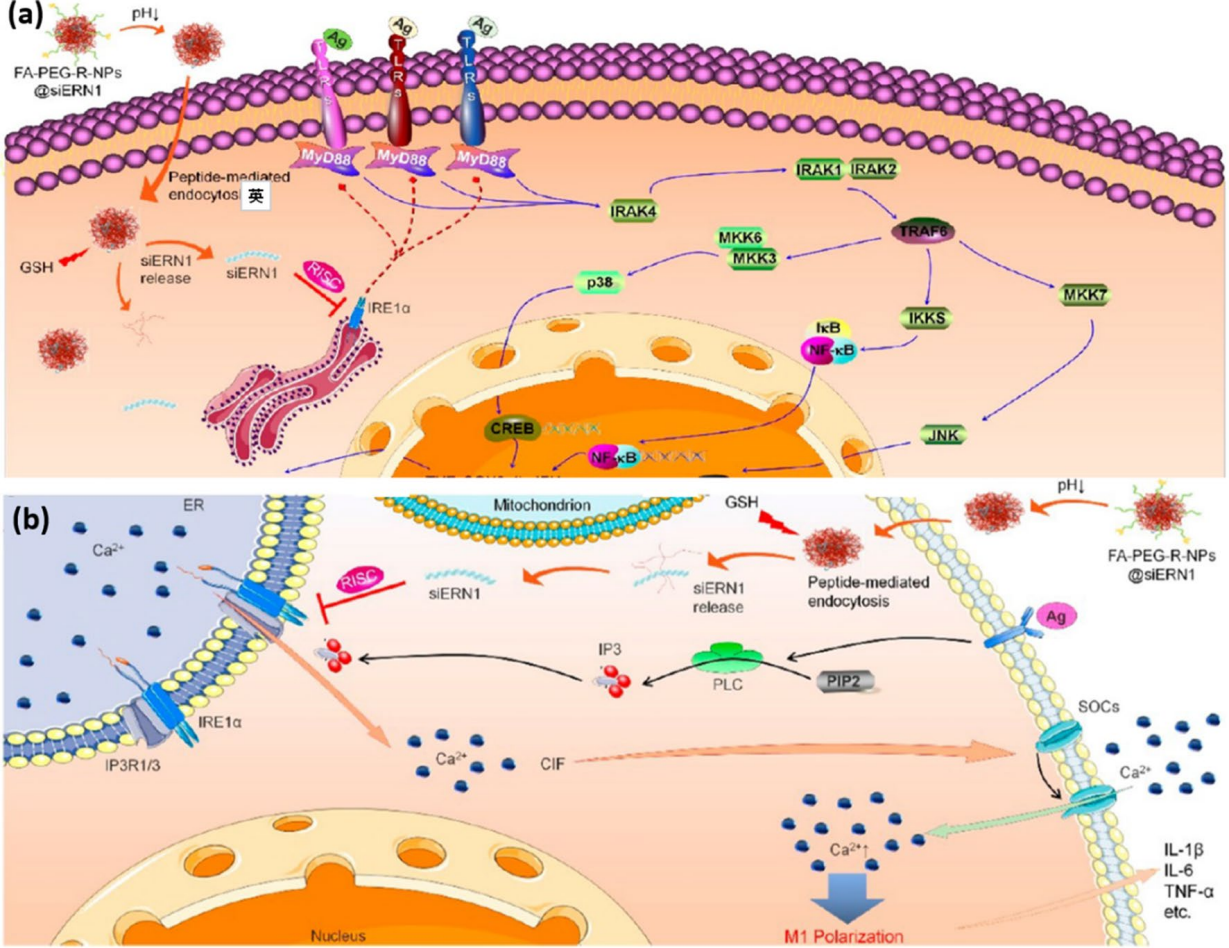
Schematic diagrams showing **a** ERN1 as an inhibitor of MyD88-dependent TLR pathway that regulates the expression of cell pro-inflammatory cytokine; **b** IRE1α as a regulator to control the intracellular concentration of Ca^2+^ ion and macrophage polarization. Reproduced with permission from reference [230]

Other than the nanocarriers that possess inherent immunomodulatory properties, some mannosylated-functionalized nanocarriers can also be applied for the delivery of immunomodulators. For example, Chaubey et al. designed a curcumin-loaded mannosylated chitosan nanoparticles and investigated its application in the treatment of visceral leishmaniasis. Curcumin is a potential modulator of M2 macrophage polarization. The nano-system successfully prolonged the retention time of curcumin and significantly enhanced its uptake by macrophages [262]. On the other hand, the use of folic acid for targeting macrophage has also been reported [263]. Notable examples include the macrophage-targeting nanomedicine FA − PEG − R − NPs@siERN1 developed by Feng and the co-workers [230]. In this study, an endoplasmic reticulum to nucleus signaling 1 gene-targeting small interfering RNA (siERN1) that possess promising inhibitory activities against the expression of pro-inflammatory cytokines was identified. The cationic core (NPs) synthesized using two cationic polymers (i.e. PEI and PBAA) was coupled to a cell-penetrating peptide RKKRRQRRR (R). Upon siERN1 loading, the core was PEG-modified (PEG − R − NPs@siERN1) to enhance the overall biocompatibility. Finally, folic acid (FA), a macrophage targeting ligand, was grafted through the PEG shell to yield the final nanomedicine. FA − PEG − R − NPs@siERN1 is an inhibitor of MyD88-dependent TLR signaling pathway and can induce M2 polarization of macrophages by regulating the concentration of calcium ion (Fig. 4). The result shows that the developed siERN1 could downregulate the expression of MyD88-dependent TLR signaling molecules and upregulate the mRNA expression of M2 macrophage markers (e.g. CD206 and Arg). The siERN1-nanoprodrug has therapeutic effects on mouse models having inflammatory bowel disease and collagen-induced arthritis (CIA). More importantly, the nano prodrug did not increase the susceptibility to bacterial infection in the CIA mouse model. This study may open up a new avenue for the development of similar strategies for the treatment of infection-associated inflammatory diseases.

Furthermore, alhagi honey polysaccharide (APH), a polysaccharide extracted from *Alhagi pseudalhagi syn*, has also been widely used as an immunomodulator. The uses of APH-loaded NPs as vaccine adjuvants have been reported. For example, Wusiman et al. reported an APH-encapsulated PLGA nanoparticles (AHPP) and found that the nanoparticles could significantly stimulate the phagocytic activity of macrophages and upregulated the Th-associated cytokines [264]. The same group also developed three kinds of APH-loaded PLGA nanoparticles modified with cationic polymers chitosan (CS-AHPP), PEI (PEI-AHPP) and ε-Poly-L-lysine (εPL-AHPP) and compared their immunomodulatory effect on macrophages [265]. The nanoparticles not only stimulated the phagocytic activities, CD80^+^ and CD86^+^ expression of macrophages, but also enhanced the level of cytokines such as IL-12, IL-1β, IFN-γ, TNF-α, with the PEI-modified nanoparticles exhibited the best stimulatory effect [266]. The result shows that surface modification with cationic polymers could effectively enhance the loading efficiency and stability of PLGA. A similar study conducted by the same group suggested that the AHP and ovalbumin loaded PEI-modified PLGA nanoparticles (PEI-AHPP/OVA) could induce the highest immune responses compared to the CS-AHPP/OVA and εPL-AHPP/OVA [267]. More recently, this research group demonstrated that the H5N1-loaded PEI-modified PLGA nanoparticles could effectively induce the production of cytokines in lymph nodes [268]. Apart from modifying with cationic polymers, Pan et al. reported an amino-modified polymer nanoparticles as an adjuvant to improve the vaccine efficacy [269]. Similar to cationic polymers, the positively charged amino group bind with the negatively charged protein e.g. ovalbumin electrostatically to form a stable complex. The formulation was found to increase the secretion of cytokines e.g. IFN-γ, TNF-α and IL-12.

On the other hand, studies using itaconate (ITA), a molecule that possess innate immunoregulatory and antioxidant effects, for macrophage immunomodulation have also been reported [270]. However, the potential of ITA is limited when it is delivered in soluble form. When taken orally, ITA is rapidly removed from circulation within 24 h. Most recently, Huyer et al. incorporated ITA into the polyester polymer backbone and successfully developed a biomimetic material with inherent immunoregulatory behavior [271]. Upon hydrolytic degradation, the ITA-polymeric material slowly releases ITA from the polyester backbones, inducing polarization of macrophage. Functional assay further reveals that the released ITA could effectively inhibit the growth of bacteria on acetate. Compared to the control materials, intraperitoneal injection of ITA polymers could rapidly resolve inflammation in mouse model.

## Summary and perspective

PRRs signaling (e.g. TLRs, NLR, and STING signaling) and innate immune cells (e.g. macrophage) are important first-line host defense systems of the body. They recognize PAMPs and/or DAMPs that gain access to the host system, and play a fundamental role in linking the innate and adaptive immunity. Increasing evidence has demonstrated the importance of PRRs and innate immune cells in the management of infectious diseases and their associated complications. While TLR-dependent signaling enables protective innate immune response, sustained and overwhelming activation of TLRs may disrupt immune homeostasis and contribute to inflammatory disorders. Recently, a range of innovative and potential TLR antagonists have been reported. Despite experiment successes, the number of TLR antagonists at clinical stage are very limited and their applications for blockage of sepsis mediators failed to show satisfactory outcome in multiple clinical trials. Learning from the prior experiences, safety and efficacy of these therapeutic agents should be comprehensively considered before entering clinical stage. Moreover, PAMPs often involve activation of multiple PRRs. Therefore, exploration of immunomodulators that target multiple PRRs may perhaps be a promising strategy for the treatment of infectious diseases. For example, Tukhvatulin and co-workers co-encapsulated NOD2 and TLR4 agonists in polymeric nanoparticles and found that the combined stimulation of NOD2 and TLR4 receptors allow synergistic activation of AP-1/NF-κB-dependent transcription.

Furthermore, various types of nanocarriers, such as liposomes, micelles, polymeric nanoparticles, metallic nanoparticles, lipid nanoparticles, dendrimers, have been commonly used for active or passive delivery of immunomodulators. Compared to free immunomodulators, nanocarrier-assisted approaches generally offer advantages of (1) higher solubility, (2) higher stability, (3) better pharmacokinetics and pharmacodynamics. Based on the nano approaches summarized in this review, incorporation/encapsulation of immunomodulators on/within nanocarriers can significantly improve the efficacy of vaccines/therapeutics. However, upon endocytosis, the fate of nanocarriers is still uncertain. For example, it has been estimated that only a small amount (less than 2%) of siRNA cargo administered via LNPs can escape the endosomes and reach the cytosol. We believe that future studies will focus more on understanding the fate of nanocarriers in cells, as well as the development of target-specific nanocarriers that enable specific delivery of immunomodulators to the target site.
